# Bootstrap-based inferential improvements to the simplex nonlinear regression model

**DOI:** 10.1371/journal.pone.0272512

**Published:** 2022-08-09

**Authors:** Alisson de Oliveira Silva, Jonas Weverson de Ararújo Silva, Patrícia L. Espinheira

**Affiliations:** 1 Instituto Federal de Educação, Ciência e Tecnologia da Paraíba, João Pessoa, Brazil; 2 Centro de Ciências Agrárias, Departamento de Ciências Fundamentais e Sociais, Areia, Paraíba, Brazil; 3 Departamento de Estatística, Universidade Federal de Pernambuco, Recife, Pernambuco, Brazil; Utrecht University: Universiteit Utrecht, NETHERLANDS

## Abstract

In this paper we evaluate the performance of point and interval estimators based on the maximum likelihood(ML) method for the nonlinear simplex regression model. Inferences based on traditional maximum likelihood estimation have good asymptotic properties, but their performance in small samples may not be satisfactory. At out set we consider the maximum likelihood estimation for the parameters of the nonlinear simplex regression model, and so we introduced a bootstrap-based correction for such estimators of this model. We also develop the percentile and bootstrap_*t*_ confidence intervals for those parameters as competitors to the traditional approximate confidence interval based on the asymptotic normality of the maximum likelihood estimators (MLEs). We then numerically evaluate the performance of these different methods for estimating the simplex regression model. The numerical evidence favors inference based on the bootstrap method, in special the bootstrap_*t*_ interval, which was decisive in an application to real data.

## 1 Introduction

Normal linear regression models are widely used in the most diverse areas of knowledge. Currently, several proposals of regression models for doubly-constrained regression models for doubly-constrained response variables, which assume continuous values in (*a*, *b*), where *a* and *b* are known and −∞ < *a* < *b* < ∞, thus, such support can be easily transformed to the unit interval.

In this context, where *y* ∈ (0, 1) or (*y* ∈ (*a*, *b*)), the normal linear model is inadequate, because besides the possibility of occurrence of fitted values smaller than 0(*a*) or larger than 1(*b*), in general, the data present asymmetry and heteroscedasticity, violating the usual assumptions of such model. Thus, it seems more appropriate to consider models based on distributions naturally supported on (0, 1) as is the case of the simplex regression model proposed by [1], for example.

The simplex distribution was developed from the generalized inverse Gaussian distribution and is part of the class of dispersion models defined by [2], which extend the [3] generalized linear models. Several papers have been conducted using this distribution. For example, [4] used it to evaluate longitudinal data considering the constant dispersion parameter, using generalized estimating equations. [5] modified this approach with the assumption that the dispersion parameter varies across observations. Based on the dispersion models, Using the Bayesian approach and Monte Carlo simulations, [6] evaluates the estimators of the parameters of the simplex model with variable dispersion.

Other approaches for modeling limited data are the beta [7], Kumaraswamy [8], Johnson *S*_*B*_ [9], unit gamma [10] regression models. Recently published papers show possible advantages of using the latter distribution over the beta distribution [11, 12]. Recently, [13] proposed to the class of non-linear simplex regression models, in which they estimate the model parameters using the maximum likelihood method and derive the local influence quantities. The authors showed that when data are concentrated at the extremes of the standard unit interval, the maximum likelihood estimation process of the simplex model is more stable than that of the beta regression model. [14] presented the zero-and-one-inflated simplex distribution for modeling proportion data. The authors introduced a new algorithm to compute maximum likelihood estimates of the parameters of the simplex distribution without covariates, and developed likelihood-based inference methods for the regression model using this new distribution.

The study of the behavior of asymptotic maximum likelihood estimators in small samples is an important area of research. These estimators can be biased when the sample size is small or even moderate. The bias is actually a measure of average risk. The average risk in replacing the true value of the parameter with a plausible estimated value. Bias can also be seen as how far the mean of an estimator is far from the true value of the parameter. Thus, it is desirable to obtain estimators with reduced bias in finite samples. When the sample size is large the bias tends to zero. In the literature, there are several ways to obtain less biased estimators in small samples. Here, we shall adopt a bias correction obtained from the bootstrap method [15].

In statistical inference it is of fundamental importance to associate reliability to the point estimates of the model, and one way to do this is through the construction of the interval estimators of the parameters in association with the probability that the estimators contain the true value of these parameters. Confidence intervals can be obtained through the assumption that the asymptotic distribution of the maximum likelihood estimators is the normal probability distribution, which may require large samples to ensure the validity of these approximation. In small samples, an alternative for constructing confidence intervals with good performance with respect to both the coverage rate of the true value of the parameter and the length of the interval is the bootstrap method [15]. Specifically we shall adopt two bootstrap-based confidence intervals, namely: the percentile and bootstrap_*t*_. These two schemes typically have empirical coverage rates very close to the nominal ones [16].

Regarding modeling limited continuous data, several authors have already conducted improvements on inference based on the maximum likelihood estimation method. [17] propose both the nonlinear beta regression model and improvements for the maximum likelihood estimators. [18] present corrections to the generalized likelihood ratio statistic (*LR*) based on [19] for the class of beta regression models whereas [11] used the same strategy considering the unitary gamma distribution. [20] also evaluate the impact of model misspecification on empirical coverage of different prediction intervals, and investigate the impact of model misspecification on three bootstrap prediction intervals. [21] discuss test inference in small samples in the class of beta regression models. The authors consider the *LR* test and its bootstrap versions, show that the standard *LR* test tends to be quite liberal in small samples and that bootstrap-based tests provide more reliable inference even when the sample size is very small.

In this document our aim is twofold. At outset we shall developed bootstrap-based inferential improvements for the parameters that index the class of nonlinear simplex regression models proposed by [13]. In which the mean of the response variable and the dispersion parameter are related to covariates by means of nonlinear predictors. In sequence, we shall jointly evaluate the performance of the competing estimators, namely: the MLEs and the bootstrap-based estimators introduced by us.

We evaluate several aspects of interval estimation by Monte Carlo simulations. The bootstrap method proved to be an important tool estimation on nonlinear simplex regression, because through it we can get around several inferential MLEs’ problems in finite samples. Finally, we present an application whose data is from the Chemistry department of the National University of Colombia.

## 2 Nonlinear simplex regression model

In the literature there are several discrete and continuous distributions that belong to the class of dispersion models, among which we can mention the distributions: normal, inverse normal, gamma, Von Mises, Poisson, Binomial, negative Binomial, and others. In particular, if a random variable *y* follows the simplex distribution denoted by *S*^−^(*μ*, *σ*^2^) with parameters 0 < *μ* < 1 and *σ*^2^ > 0, the density expression takes the following form:

$$\begin{array}{c} p\left(y;\mu ,\sigma^{2}\right)={\left[2\pi \sigma^{2}{\left\{y\left(1-y\right)\right\}}^{3}\right]}^{-1/2}\text{exp}\{-\frac{1}{2\sigma^{2}}d\left(y;\mu \right)\},\;y\in \left(0,1\right), \end{array}$$ (1)

where the deviance component *d*(*y*; *μ*) is given by $d\left(y;\mu \right)=\frac{{\left(y-\mu \right)}^{2}}{y\left(1-y\right)\mu^{2}{\left(1-\mu \right)}^{2}}.$ The variance function for the simplex distribution is expressed as *V*(*μ*) = *μ*^3^(1 − *μ*)^3^. The mean and variance of this distribution are given, respectively, by E(y)=μ and $\text{Var}\left(y\right)=\mu \left(1-\mu \right)-\sqrt{\frac{1}{2\sigma^{2}}}\;\text{exp}\;\{\frac{1}{\sigma^{2}\mu^{2}{\left(1-\mu \right)}^{2}}\}\Gamma (\frac{1}{2},\frac{1}{2\sigma^{2}\mu^{2}{\left(1-\mu \right)}^{2}}),$ where Γ(*a*, *b*) corresponds to the incomplete gamma function, defined by $\Gamma \left(a,b\right)=\int_{b}^{\infty }x^{a-1}e^{-x}dx$. For more details on these properties, see [2]. The simplex distribution is quite flexible for modeling data in the continuous range (0, 1), showing different shapes according to the values of the parameters that index the distribution. For examples, such as the *J* shape for *S*^−^(0.9, 36), the *U* shape for *S*^−^(0.5, 121) and the inverse J shape for *S*^−^(0.1, 36), in addition to the common shapes, namely left-symmetric, right-symmetric and symmetric. Also, unlike the beta distribution, the simplex model is very useful for accommodating data with bimodal distributions, example for *S*^−^(0.5, 20).

Let *y*_1_, …, *y*_*n*_ be independent random variables, where each *y*_*t*_, *t* = 1, …, *n*, follows a simplex distribution, whose probability density function is given by (1) with mean *μ*_*t*_ and dispersion parameter $\sigma_{t}^{2}$. The nonlinear simplex regression model proposed by [13] is defined by (1) and the systematic components given by

$$\begin{array}{c} g\left(\mu_{t}\right)=f_{1}\left(x_{t}^{⊤};\beta \right)=\eta_{t}\;\text{and}\;h\left(\sigma_{t}^{2}\right)=f_{2}\left(z_{t}^{⊤};\gamma \right)=\zeta_{t},\;t=1,\ldots ,n, \end{array}$$ (2)

where *β* = (*β*_1_, …, *β*_*k*_)^⊤^ and *γ* = (*γ*_1_, …, *γ*_*q*_)^⊤^ are unknown regression parameter vectors such that $\beta \in R^{k}$ and $\gamma \in R^{q}$, *k* + *q* < *n*, *η*_*t*_ = (*η*_1_, …, *η*_*n*_)^⊤^ and *ζ*_*t*_ = (*ζ*_1_, …, *ζ*_*n*_)^⊤^ are nonlinear predictors and $x_{t}^{⊤}=\left(x_{t1},\ldots ,x_{tk_{1}}\right)$ and $z_{t}^{⊤}=\left(z_{t1},\ldots ,z_{tq_{1}}\right)$ are, respectively, *k*_1_ and *q*_1_ observations of known covariates, which may coincide fully or partially such that *k*_1_ ≤ *k* and *q*_1_ ≤ *q*.

In the linear models *k*_1_ = *k*, *q*_1_ = *q*, and therefore, $x_{t}^{⊤}=\left(x_{t1},\ldots ,x_{tk}\right)$ and $z_{t}^{⊤}=\left(z_{t1},\ldots ,z_{tq}\right)$ are, respectively, the *t*-th rows of the matrices *X* and *Z*, for *t* = 1, …, *n*. Linear models are a particular case of nonlinear models. When we have nonlinearity in the parameters, at least one of the ∂*f*_1_(⋅; *β*)/∂*β*_*j*_, *j* = 1, …, *k* depends on *β*^⊤^ = (*β*_1_, …, *β*_*k*_) and, at least one of the ∂*f*_2_(⋅; *γ*)/∂ *γ*_*l*_, *l* = 1, …, *q* depends on *γ*^⊤^ = (*γ*_1_, …, *γ*_*q*_). For linear simplex models, these derivatives depend only on the covariates *x*_1_, …, *x*_*k*_ and *z*_1_, …, *z*_*q*_, respectively, and so $\tilde{X}=\partial \eta /\partial \beta =X$ and $\tilde{Z}=\partial \zeta /\partial \gamma =Z$.

Moreover in (2) the link functions g:(0,1)→R and h:(0,∞)→R are strictly monotone and at least twice differentiable. Different link functions can be chosen for *g* and *h*. For example, for *μ* we can use the logit function *g*(*μ*) = log{*μ*/(1 − *μ*)}, the probit function *g*(*μ*) = Φ^−1^(*μ*), where Φ(⋅) denotes the standard normal distribution function, the log-log function *g*(*μ*) = log{−log(1 − *μ*)} and the log-log complementary function *g*(*μ*) = log{−log(1 − *μ*)}, among others. Since *σ*^2^ > 0, we can use the log function *h*(*σ*^2^) = log(*σ*^2^) and the identity function *h*(*σ*^2^) = *σ*^2^. However, one should be aware if the estimates resulting from the likelihood maximization process take on positive values. If, in fact, the identity link function is the most appropriate, negative estimates shall not occur for the $\sigma_{t}^{2}$, *t* = 1, …, *n* and the diagnostic analysis shall corroborate for the model goodness-of-fit to the data when using such a link function. For more details, see [3, 22]. Finally, in (2) we have that *g*(*μ*_*t*_) = *η*_*t*_ and $h\left(\sigma_{t}^{2}\right)=\zeta_{t}$, *t* = 1, …, *n*, are the mean and the dispersion submodels, respectively.

To provide the quantities related to the estimation by the maximum likelihood procedure we shall consider the general case, with nonlinearity in the parameters. Thus, we must emphasize that, *f*_1_(⋅) and *f*_2_(⋅) are differentiable functions with Jacobian matrices. Based on (1) we have that the logarithm of the likelihood function is given by $\ell \left(\beta ,\gamma \right)=\sum_{t=1}^{n}\ell_{t}\left(\mu_{t},\sigma_{t}^{2}\right)$, in which

$$\begin{array}{c} \ell_{t}\left(\mu_{t},\sigma_{t}^{2}\right)=-\frac{1}{2}\;\text{log}\;2\pi -\frac{1}{2}\;\text{log}\;\sigma_{t}^{2}-\frac{3}{2}\;\text{log}\;\left\{y_{t}\left(1-y_{t}\right)\right\}-\frac{1}{2\sigma_{t}^{2}}d\left(y_{t};\mu_{t}\right). \end{array}$$

The components of the score vector (*U*_*β*_(*β*, *γ*)^⊤^, *U*_*γ*_(*β*, *γ*)^⊤^)^⊤^ are given by $U_{\beta }\left(\beta ,\gamma \right)={\tilde{X}}^{⊤}SUT\left(y-\mu \right)\;\text{and}\;U_{\gamma }\left(\beta ,\gamma \right)={\tilde{Z}}^{⊤}Ha,$ with $\tilde{X}=\frac{\partial \eta }{\partial \beta }$ and $\tilde{Z}=\frac{\partial \zeta }{\partial \gamma }$ being derivative matrices of dimension *n* × *k* and *n* × *q*, respectively, *y* = (*y*_1_, …, *y*_*n*_)^⊤^, *μ* = (*μ*_1_, …, *μ*_*n*_)^⊤^ and *a* = (*a*_1_, …, *a*_*n*_)^⊤^ are *n* × 1 matrices and *U* = diag{*u*_1_, …, *u*_*n*_} is a diagonal matrix in which the *t*-th component is defined as

$$\begin{array}{c} u_{t}=\frac{1}{\mu_{t}\left(1-\mu_{t}\right)}\{d\left(y_{t};\mu_{t}\right)+\frac{1}{\mu_{t}^{2}{\left(1-\mu_{t}\right)}^{2}}\}\;\text{with}\;a_{t}=-\frac{1}{2\sigma_{t}^{2}}+\frac{d(y_{t};\mu_{t})}{2{\left(\sigma_{t}^{2}\right)}^{2}}. \end{array}$$

Moreover, $S=\text{diag}\{\sigma_{1}^{2},\ldots ,\sigma_{n}^{2}\}$

$$\begin{array}{c} T=\text{diag}\{\frac{1}{g^{\prime }\left(\mu_{1}\right)},\ldots ,\frac{1}{g^{\prime }\left(\mu_{n}\right)}\}\;\text{and}\;H=\text{diag}\{\frac{1}{h^{\prime }\left(\sigma_{1}^{2}\right)},\ldots ,\frac{1}{h^{\prime }\left(\sigma_{n}^{2}\right)}\}. \end{array}$$ (3)

To obtain the Fisher information matrix for the parameter vectors *β* and *γ*, we shall use the following results: $E\left[d\left(y;\mu \right)\right]=\sigma^{2}$, $E\left[\left(y-\mu \right)d^{\prime }\left(y;\mu \right)\right]=-2\sigma^{2}$ and E[(y-μ)d(y;μ)]=0 [4]; $E[\left(y-\mu \right)d^{\prime \prime }\left(y;\mu \right)]=0$, $\frac{1}{2}E\left[d^{\prime \prime }\left(y;\mu \right)\right]=\frac{3\sigma^{2}}{\mu (1-\mu )}+\frac{1}{\mu^{3}{\left(1-\mu \right)}^{3}}$ and Var[*d*(*y*; *μ*)] = 2(*σ*^2^)^2^ [23] and $E[d^{\prime }\left(y;\mu \right)]=0$ [5]. The Fisher information matrix for the parameter vector *θ* = (*β*^⊤^, *γ*^⊤^)^⊤^ so-called here by *K*(*β*, *γ*) is a diagonal matrix with two blocks of submatrices which are *K*_*ββ*_ and *K*_*γγ*_ defined as follows $K_{\beta \beta }={\tilde{X}}^{⊤}SW\tilde{X}$ and $K_{\gamma \gamma }={\tilde{Z}}^{⊤}D\tilde{Z}$. Here, *W* = diag{*w*_1_, …, *w*_*n*_} and *D* = diag(*d*_1_, …, *d*_*n*_) with

$$\begin{array}{c} w_{t}=(\frac{3\sigma_{t}^{2}}{\mu_{t}\left(1-\mu_{t}\right)}+\frac{1}{\mu_{t}^{3}{\left(1-\mu_{t}\right)}^{3}})\frac{1}{{\left[g^{{}^{\prime }}\left(\mu_{t}\right)\right]}^{2}}\;\text{and}\;d_{t}=\frac{1}{2{\left(\sigma_{t}^{2}\right)}^{2}}\frac{1}{{\left[h^{{}^{\prime }}\left(\sigma_{t}^{2}\right)\right]}^{2}}. \end{array}$$

Since *K*(*β*, *γ*) is a diagonal block matrix, the vectors *β* and *γ* are globally orthogonal [24] so that their MLEs $\hat{\beta }$ and $\hat{\gamma }$ are asymptotically independent. For large samples and under regularity conditions the approximate distribution of the MLEs is given by

$$\begin{array}{c} (\begin{array}{c} \hat{\beta }\\ \hat{\gamma } \end{array})\approx N_{k+q}((\begin{array}{c} \beta \\ \gamma \end{array}),K^{-1})\;\text{and}\;K^{-1}=(\begin{array}{cc} {\left({\tilde{X}}^{⊤}SW\tilde{X}\right)}^{-1} & 0\\ 0 & {\left({\tilde{Z}}^{⊤}D\tilde{Z}\right)}^{-1} \end{array}). \end{array}$$ (4)

To measure the degree of non-constant dispersion, we define $\lambda =\frac{\sigma_{t\text{max}}^{2}}{\sigma_{t\text{min}}^{2}}$, *t* = 1, …, *n*. Note that the greater the λ the further away the simplex regression model with varying dispersion is from the model in which the dispersion is supposed to be fixed, since the **constant dispersion models** holds that $\sigma_{1}^{2}=\ldots =\sigma_{n}^{2}=\sigma^{2}$, in either case λ = 1. Furthermore, this λ definition measure actually as the increase of variance response effects the estimation process of the model. To became *σ*^2^ variable it is necessary increases the $\sigma_{\text{max}}^{2}$, otherwise the $\sigma_{\text{min}}^{2}$ should be too small and, do not plausible. Thus, as greater is λ as greater is the response variances, in the real problems. Here during the simulations we control the value of the maximum variance, because exactly what we want is to evaluate the properties of the estimators when the variance does not explode, but only grows slightly. When working with real data, the occurrence of large values of estimated λ is substantial, i.e., λ > 1 (in particular, *n* when it is large).

We still need to discuss the variances of the responses further. The first part of the expression in (4) implies that the vector ${\left({\hat{\beta }}^{⊤},{\hat{\gamma }}^{⊤}\right)}^{⊤}$ is *asymptotically* unbiased. Thus, as the sample size increases, ${\left({\hat{\beta }}^{⊤},{\hat{\gamma }}^{⊤}\right)}^{⊤}$ is *approximately* unbiased and its bias should be close to zero. In theory this fact is true only when *n* approaches infinity, that is, asymptotically. In practice the better the approximation in (4), and this depends on the distribution, the faster the bias goes to zero, i.e. this can occur for sample sizes *n* = 40, 50….

However, this assumption is mostly valid for ${\hat{\beta }}^{⊤}$ due to its relationship with $\hat{\mu }$ which is theoretically unbiased, (exactly and not approximately, typically). On the other hand, the relationship of the ${\hat{\gamma }}^{⊤}$ vector is with ${\hat{\sigma }}^{2}$, which is theoretically biased in most distributions. Thus, it is already expected that the ${\hat{\gamma }}^{⊤}$ bias takes a long time to converge to zero and requires large sample sizes for this to occur.

This discussion reveals that we should be more aware of how the corrections act on the ${\hat{\gamma }}^{⊤}$. Note that biased ${\hat{\gamma }}^{⊤}$ shall induce biased response variances. As a consequence, hypothesis tests and confidence intervals should perform poorly and may lead to misleading conclusions about the model, such as the exclusion of important covariates.

## 3 Point estimation of the model parameters

The maximum likelihood estimators of *β* and *γ* are obtained by Fisher’s interactive scoring process in which the initial guess was proposed in [13], and are usually biased when the sample size is small or even moderate, particularly ${\hat{\gamma }}^{⊤}$. Nevertheless, the estimator’s bias can be corrected and one possibility is to use resampling methods, which are schemes that use repeated sampling within the same sample to calculate estimates. The bootstrap method is one of the most widely used resampling methods, and one that gives very satisfactory results for estimating a model. In this paper we adopt the parametric bootstrap where in the regression models context it assumes that the probability distribution of the response variable is known and indexed by unknown parameters. [15]. The steps for performing this method both for bias correction of the MLEs and for obtaining the confidence interval are described in the Algorithms (1), (2) and (3).

**Algorothm 1**: Parametric method

1: Suppose that *y* = (*y*_1_, …, *y*_*n*_)^⊤^ is a random sample such that each *y*_*t*_, *t* = 1, …, *n*, follows a distribution *F* supposedly known and indexed by parameter vector *θ*;

2: From the original sample, obtain the $\hat{\theta }$ estimate of *θ*;

3: Generate *B* bootstrap samples of size *n*, namely $y_{b}^{*}=\left(y_{1}^{*},\ldots ,y_{n}^{*}\right)$ from $F(\hat{\theta })$, *b* = 1, …, *B*;

4: For each bootstrap sample $y_{b}^{*}$ compute ${\hat{\theta }}^{*b}$;

5: Repeat steps 3 and 4 a great number *B* of times, thus obtaining ${\hat{\theta }}^{*1},\ldots ,{\hat{\theta }}^{*B}$;

6: Use the estimates ${\hat{\theta }}^{*b}$, with *b* = 1, …, *B* for compute the desired quantities, for instance: mean, variance, confidence interval, etc. regarding distribution of *y*.

Once the estimate of the estimator’s bias is obtained we can construct the bias-corrected point estimators. Using the steps of the bootstrap method presented in Algorithm (1), a bootstrap estimate of the bias can be obtained by

$$\begin{array}{c} {\hat{B}}_{boot}\left(\hat{\theta }\right)={\bar{\theta }}^{*}-\hat{\theta }, \end{array}$$

where ${\bar{\theta }}^{*}=\frac{1}{B}\sum_{b=1}^{B}{\hat{\theta }}^{*b}$, i.e., it is possible to approximate the expected value from the arithmetic mean of the bootstrap estimates of *θ*. Thus, we can obtain an estimator corrected up to second order by bootstrap [15, 25]:

$$\begin{array}{c} \bar{\theta }=\hat{\theta }-{\hat{B}}_{boot}\left(\hat{\theta }\right)=\hat{\theta }-\left({\bar{\theta }}^{*}-\hat{\theta }\right)=2\hat{\theta }-{\bar{\theta }}^{*}. \end{array}$$

This estimator has the same asymptotic properties as the usual MLE and presents Lower bias in small samples [16]. A detailed discussion of the bootstrap second-order bias correction and its relation to the analytic correction can be found in [26].

## 4 Interval estimation of the model parameters

A set constructed on the basis of a point estimator in association with a probability that this set contains the true value of the parameter, defines a confidence interval estimator. The general form for approximate confidence intervals (CI) for *θ* is: $P[l_{1}\leq \theta \leq l_{2}]\approx 1-\alpha ,\;0<\alpha <1,$ where *l*_1_ and *l*_2_, (*l*_1_ < *l*_2_) are the lower and upper bounds of the confidence interval, respectively, and 1 − *α* is the confidence level which converges to the probability of coverage. We should emphasize that *l*_1_ and *l*_2_ are quantiles of a distribution indexed by the parameter *θ*. Whether we assume that this distribution is known, it is possible to construct exact confidence intervals. However, defining the exact analytical distribution of a random variable is typically highly challenging.

Fortunately, there are diverse approaches to building approximate confidence intervals. The most widely used is the asymptotic confidence interval, which assumes asymptotic normality of the MLEs. According to (4) for the simplex model in large samples the distribution of $\hat{\theta }={\left({\hat{\beta }}^{⊤},{\hat{\gamma }}^{⊤}\right)}^{⊤}$ is approximately normal with mean equal to *θ* = (*β*^⊤^, *γ*^⊤^)^⊤^ and the variance and covariance matrix given by (4). More precisely, we have that $K^{\beta }={\left({\tilde{X}}^{⊤}SW\tilde{X}\right)}^{-1}$ evaluated at $\hat{\theta }$ is the *k* × *k* matrix of variances and covariances of $\hat{\beta }$ and $K^{\gamma }={\left({\tilde{Z}}^{⊤}D\tilde{Z}\right)}^{-1}$ evaluated at $\hat{\theta }$ is the *q* × *q* matrix of variances and covariances of $\hat{\gamma }$.

Consider *β*_*i*_ and *γ*_*j*_, with *i* = 1, …, *k* and *j* = 1, …, *q*, the *i*th and *j*th components of the vectors *β* and *γ*, respectively. We shall denote $K_{i}^{\beta \beta }$ and $K_{j}^{\gamma \gamma }$ as the *i*-th and *j*-th components of the main diagonal of the matrices $K^{\beta \beta }\left(\hat{\theta }\right)$ and $K^{\gamma \gamma }\left(\hat{\theta }\right)$, respectively. Therefore, it follows that $\left({\hat{\beta }}_{i}-z_{1-\frac{\alpha }{2}}{\left(K_{i}^{\beta \beta }\right)}^{1/2};{\hat{\beta }}_{i}+z_{1-\frac{\alpha }{2}}{\left(K_{i}^{\beta \beta }\right)}^{1/2}\right)$ and $\left({\hat{\gamma }}_{j}-z_{1-\frac{\alpha }{2}}{\left(K_{j}^{\gamma \gamma }\right)}^{1/2};{\hat{\gamma }}_{j}+z_{1-\frac{\alpha }{2}}{\left(K_{j}^{\gamma \gamma }\right)}^{1/2}\right)$ are intervals with confidence approximately equal to 1 − *α* for *β*_*i*_ and *γ*_*j*_, respectively, where $z_{1-\frac{\alpha }{2}}$ is the $1-\frac{\alpha }{2}$ quantile of the standard normal distribution. These intervals based on MLE may require large samples for the coverage to be close to the nominal ones. In small samples, they can have large coverage errors [15, 25].

An workaround for reaching improvements to confidence intervals in small samples, without analytical complexities, is the bootstrap method. This approach typically provides confidence intervals that have coverage levels close to the true coverage probability. Here, we shall discuss two strategies bootstrap-based confidence intervals, namely: the percentile and bootstrap_*t*_.

The percentile bootstrap confidence interval we shall denote by ‘**Boot***p*’ [16] is a bootstrap approach built based on a *B* finite replicates of the estimators of the parameters of interest. Furthermore, it displays the monotonic transformation invariance property. Let *F*(*θ*) be the distribution of the response variable assumed to be known and indexed by the parameter vector *θ*. Moreover, let $\hat{G}$ be the empirical distribution function of $\hat{\theta }$ obtained from the *B* bootstrap replicas. We can construct the percentile confidence interval, with approximate coverage level 1 − *α*, by calculating 1 − *α*/2 and *α*/2 quantiles of $\hat{G}$. The interval is given by $({\hat{G}}^{-1}\left(\alpha /2\right),{\hat{G}}^{-1}\left(1-\alpha /2\right)).$ Defining ${\hat{G}}^{-1}\left(\alpha /2\right)={\hat{\theta }}^{*(\alpha /2)}$ and ${\hat{G}}^{-1}\left(1-\alpha /2\right)={\hat{\theta }}^{*(1-\alpha /2)}$. The expressions of the percentile bootstrap confidence intervals for the parameters of the nonlinear simplex regression model are given by:

$$\begin{array}{c} \left({\hat{\beta }}_{i}^{*(\alpha /2)},{\hat{\beta }}_{i}^{*(1-\alpha /2)}\right)\;\text{and}\;\left({\hat{\gamma }}_{j}^{*(\alpha /2)},{\hat{\gamma }}_{j}^{*(1-\alpha /2)}\right). \end{array}$$ (5)

*i* = 1, …, *k* and *j* = 1, …, *q*, the *i*-th and *j*-th components of the vectors *β* and *γ*. The percentile interval is not necessarily symmetric about the value of $\hat{\beta }$ and $\hat{\gamma }$. Its construction ensures that improper values for the parameter of interest are not included in the confidence interval. The steps for its construction are described in Algorithm 2.

**Algorithm 2**: Bootstrap confidence interval—Percentile

1: Generate *B* bootstrap samples $y^{*b}=\left(y_{1}^{*},y_{2}^{*},\ldots ,y_{n}^{*}\right)$ based on $F(\hat{\theta })$, for *b* = 1, …, *B*;

2: Let $\hat{\theta }=s\left(y\right)$, in which, *y* = (*y*_1_, …, *y*_*n*_) is the original sample. Thus, the respective bootstrap estimate of de *θ* is computed as follows: ${\hat{\theta }}^{*b}=s\left(y^{*b}\right)$, *b* = 1, …, *B*;

3: The *B* replicas of ${\hat{\theta }}^{*}$ must be ordered.

4: The lower and upper limits of the percentile interval are provide by the replicas of ${\hat{\theta }}^{*}$ of order *B* × (*α*/2) and *B* × (1 − *α*/2), respectively, by assuming that *B* × (*α*/2) and *B* × (1 − *α*/2) are integers and 0 < *α* < 1; Meaning ${\hat{G}}^{-1}\left(\alpha /2\right)={\hat{\theta }}^{*(\alpha /2)}$ and ${\hat{G}}^{-1}\left(1-\alpha /2\right)={\hat{\theta }}^{*(1-\alpha /2)}$.

 4.1: Whether *B* × (1 − *α*/2) and *B* × (1 − *α*/2) are not integers, we can use the following procedure:

 4.1.1: Assuming 0 < *α* < 1, let *p* = [(*B* + 1)*α*/2] be the largest integer less than or equal to the number (*B* + 1)*α*/2; then, we define the lower and upper bounds of the percentile interval by the *p*-th and (*B* + 1 − *p*)-th ordered elements of the *B* bootstrap replicas of ${\hat{\theta }}^{*}$, respectively.

The bootstrap_*t*_ confidence interval, here so-called as ‘**Boot**_*t*_’ [16] is a pivotal method to construct confidence intervals that rely on the traditional *t*-Student confidence interval. This interval is based on the bootstrap estimate of the *T* distribution, where *T* is given by

$$\begin{array}{c} T=\frac{\hat{\theta }-\theta }{\hat{ep}\left(\hat{\theta }\right)}, \end{array}$$

where $\hat{ep}\left(\hat{\theta }\right)$ is the standard error of $\hat{\theta }$. The construction of the bootstrap_*t*_ confidence interval is given by Algorithm 3.

**Algorithm 3**: Bootstrap confidence interval—Bootstrap_*t*_

1: Generate *B* bootstrap samples $y^{*b}=\left(y_{1}^{*},y_{2}^{*},\ldots ,y_{n}^{*}\right)$ from $F(\hat{\theta })$;

2: For each bootstrap sample, it is compute

$$\begin{array}{c} T^{*b}=\frac{{\hat{\theta }}^{*b}-\hat{\theta }}{{\hat{ep}}^{*b}}, \end{array}$$

with *b* = 1, 2, …, *B*, where $\hat{\theta }=s\left(y\right)$ is the estimated value of *θ* from the original sample *y*, ${\hat{\theta }}^{*b}=s\left(y^{*b}\right)$ is the estimated value of *θ* for the bootstrap sample y^*b^ and ${\hat{ep}}^{*b}$ is the standard error of ${\hat{\theta }}^{*b}$ for the bootstrap sample y^*b^. Note that *ep* = *κ*(*θ*), *κ* known function and ${\hat{ep}}^{*b}=\kappa \left({\hat{\theta }}^{*b}\right)$;

3: The *α*/2 and 1 − *α*/2 percentiles of T^*b^ are estimate by the values ${\hat{t}}^{*\left(\alpha /2\right)}$ and ${\hat{t}}^{*(1-\alpha /2)}$, respectively, as follows

$$\begin{array}{c} \frac{\#\{T^{*b}\leq {\hat{t}}^{*(\alpha /2)}\}}{B}=\frac{\alpha }{2}\;\text{and}\;\frac{\#\{T^{*b}\leq {\hat{t}}^{*(1-\alpha /2)}\}}{B}=1-\frac{\alpha }{2}. \end{array}$$

Thus, the bootstrap_*t*_ confidence interval is given by

$$\begin{array}{c} (\hat{\theta }-{\hat{t}}^{*(1-\alpha /2)}{\hat{ep}}^{*},\hat{\theta }-{\hat{t}}^{*(\alpha /2)}{\hat{ep}}^{*}), \end{array}$$

in which ${\hat{ep}}^{*}\equiv \hat{ep}\left({\hat{\theta }}^{*}\right)$. The amounts ${\hat{t}}^{*(\alpha /2)}$ and ${\hat{t}}^{*(1-\alpha /2)}$ can be obtained as follows:

**1**. Sort the *B* bootstrap replicas T^*b^;

**2**. The quantiles ${\hat{t}}^{*(\alpha /2)}$ and ${\hat{t}}^{*(1-\alpha /2)}$ are, respectively, the replicas corresponding to the integer parts of *B* × (*α*/2) and *B* × (1 − *α*/2);

**2.1**. If *B* × (*α*/2) and *B* × (1 − *α*/2) are not integers, we can use the following procedure:

Assuming 0 < *α* < 1, is *k* = [(*B* + 1)*α*/2] the largest integer less than or equal to the number (*B* + 1)*α*/2. Thus, the quantiles bootstrap ${\hat{t}}^{*(\alpha /2)}$ and ${\hat{t}}^{*(1-\alpha /2)}$ are given, respectively, by the *k*-th and (*B* + 1 − *k*)-th ordered elements of T^*b^. Therefore, the bootstrap_*t*_ intervals for the parameters of the simplex nonlinear regression model are given by the following expressions

$$\begin{array}{c} \left({\hat{\beta }}_{i}-{\hat{t}}^{*(1-\alpha /2)}{\hat{ep}}^{*},{\hat{\beta }}_{i}-{\hat{t}}^{*(\alpha /2)}{\hat{ep}}^{*}\right)\;\text{and}\;\left({\hat{\gamma }}_{j}-{\hat{t}}^{*(1-\alpha /2)}{\hat{ep}}^{*},{\hat{\gamma }}_{j}-{\hat{t}}^{*(\alpha /2)}{\hat{ep}}^{*}\right). \end{array}$$

with *i* = 1, …, *k* and *j* = 1, …, *q*, the *i*-th and *j*-th components of the vectors *β* and *γ*.

According to [16], the bootstrap_*t*_ intervals outperform the asymptotic interval displaying empirical coverages closer to the exact nominal levels, but tend not to be accurate in actual practice. Percentile intervals are more accurate, but display less satisfactory coverage performances. An outstanding discussion on bootstrap-based confidence intervals can be found in [27]. In what follows we shall evaluate the finite-sample performances of the confidence intervals introduced in this section.

## 5 Numerical results on point estimation

In this section we present the Monte Carlo simulations results, carried out to evaluate the performances of the maximum likelihood estimators of the nonlinear simplex regression model and the bootstrap versions on small samples. In what follows we shall assuming the following nonlinear simplex regression model:

$$\begin{array}{c} g\left(\mu_{t}\right)=\beta_{1}+x_{t2}^{\beta_{2}}+\beta_{3}x_{t3}+\beta_{4}x_{t4}\;\text{and}\;h\left(\sigma_{t}^{2}\right)=\gamma_{1}+z_{t}^{\gamma_{2}},\;t=1,\ldots ,n, \end{array}$$ (6)

where *g*(⋅) and *h*(⋅) are the logit and logarithmic link functions, respectively. The realizations of the covariates were generated using the uniform distribution as follows: $x_{t2}∼U\left(0.5,1.5\right)$, $x_{t3}∼U\left(0,1\right)$, $x_{t4}∼U\left(-0.5,0.5\right)$ and $z_{t}∼U\left(0.5,1.5\right)$ which are retained fixed for each Monte Carlo replication. Three different scenarios were considered for the mean response, namely: *μ*_*t*_ ∈ (0.02, 0.32) with *β* = (−2.4, 1.2, −1.5, −1.7)^⊤^; *μ*_*t*_ ∈ (0.19, 0.86) with *β* = (−1.7, −1.8, 1.2, −1.3)^⊤^ and *μ*_*t*_ ∈ (0.78, 0.98) with *β* = (2.1, −1.5, −1.6, −1.2)^⊤^. Furthermore, concerning the degree of non-constant dispersion, we report here the results for λ ≈ 12 with *γ* = (−1.3, −1.6)^⊤^; λ ≈ 45 with *γ* = (−1.3, −2.1)^⊤^ and λ ≈ 128 with *γ* = (−1.3, −2.4)^⊤^. The sample sizes chosen were *n* = 40, 80 and 120. For the last two cases we initially generated *n* = 40 covariates observations and these were replicated twice and three times, respectively to obtain the sample sizes *n* = 80 and *n* = 120. This was done to ensure that the non-constant dispersion intensity was the same for all sample sizes. The number of Monte Carlo and bootstrap replications were *R* = 10000 and *B* = 500, respectively. The parameter estimates in (6) were obtained by maximizing the log-likelihood function using the Fisher’s nonlinear optimization method.

For each both Monte Carlo replicate and the maximum likelihood estimate of the model parameters, *B* bootstrap replicate estimates were generated. Thus, at the end of the bootstrap some quantities regarding the parameters are estimated, namely: the corrected bootstrap estimates and the bootstrap confidence intervals, percentile and bootstrap_*t*_. Finally, outside the bootstrap, the asymptotic intervals of the parameters are also computed based on the quantiles of the standard normal distribution.

Aiming to evaluate the performance of the point estimation of the parameters, the relative bias and the square root of the mean square error were calculated for each sample size. Additionally, we introduce a measure suggested during the review of the article, which we shall so-call Unified Quadratic Bias (UQB) define as $\sqrt{bias_{\beta_{1}}^{2}+\ldots +bias_{\gamma_{2}}^{2}}$. In Tables 1–3 we consider, respectively, the scenarios where *μ*_*t*_ ∈ (0.02, 0.32), (*μ*_*t*_ ≈ 0), *μ*_*t*_ ∈ (0.19, 0.86), (*μ*_*t*_ ≈ 0.5) and *μ*_*t*_ ∈ (0.78, 0.98), (*μ*_*t*_ ≈ 1), *t* = 1, …, *n*. In these tables are reported the relative biases and the square roots of the mean square errors (RMSEs) of the parameter estimators for *n* = 40, 80 and 120 and λ ≈ 12, 45 and 128. We observe that in modulo the estimates of the relative bias of the bootstrap corrected estimators are smaller than those of the maximum likelihood estimators, evidencing the efficacy of the bootstrap scheme in bias correction.

**Table 1:** Relative biases and root mean square errors of the Maximum Likelihood Estimators (MLEs-asymptotic) and bootstrap corrected MLEs of the model parameters: $\text{Logit}\left(\mu_{t}/1-\mu_{t}\right)=\beta_{1}+x_{t2}^{\beta_{2}}+\beta_{3}x_{t3}+\beta_{4}x_{t4}$ and $\text{log}\left(\sigma_{t}^{2}\right)=\gamma_{1}+z_{t}^{\gamma_{2}}$, *t* = 1, …, *n*, *β* = (−2.4, 1.2, −1.5, −1.7)^⊤^, *μ*_*t*_ ∈ (0.02, 0.32), *t* = 1, …, *n*.

*μ*_*t*_ ∈ (0.02, 0.32)													
n	*θ*	λ ≈ 12				λ ≈ 45				λ ≈ 128			
		MLE		BOOT		MLE		BOOT		MLE		BOOT	
		Bias	$\sqrt{MSE}$	Bias	$\sqrt{MSE}$	Bias	$\sqrt{MSE}$	Bias	$\sqrt{MSE}$	Bias	$\sqrt{MSE}$	Bias	$\sqrt{MSE}$
40	*β*_1_	0.0003	0.082	−0.001	0.082	0.001	0.085	0.0002	0.0854	0.002	0.089	0.001	0.089
	*β*_2_	−0.001	0.131	0.001	0.131	0.004	0.134	0.004	0.135	0.004	0.143	0.002	0.142
	*β*_3_	0.003	0.137	0.002	0.138	0.001	0.143	0.0003	0.143	0.001	0.153	−0.0002	0.153
	*β*_4_	−0.001	0.115	−0.001	0.115	−0.001	0.116	−0.001	0.116	0.001	0.123	0.001	0.123
	*γ*_1_	0.136	0.274	−0.001	0.239	0.136	0.273	−0.001	0.237	0.138	0.271	0.003	0.233
	*γ*_2_	−0.010	0.323	0.001	0.311	<0.0001	0.233	0.001	0.229	0.001	0.191	0.001	0.188
UQB		0.136		0.003		0.136		0.004		0.138		0.004	
80	*β*_1_	0.001	0.057	0.001	0.057	<0.0001	0.059	−0.001	0.059	0.001	0.064	0.0003	0.064
	*β*_2_	−0.001	0.091	<0.0001	0.091	0.002	0.094	0.001	0.095	0.003	0.099	−0.001	0.098
	*β*_3_	−0.001	0.093	−0.001	0.093	0.002	0.098	0.001	0.098	<0.0001	0.108	−0.001	0.108
	*β*_4_	−0.0004	0.079	−0.0004	0.079	−0.0002	0.081	<0.0001	0.081	−0.001	0.085	−0.001	0.085
	*γ*_1_	0.067	0.173	0.002	0.160	0.065	0.173	0.001	0.162	0.060	0.168	−0.001	0.158
	*γ*_2_	−0.0004	0.209	0.003	0.206	−0.0004	0.154	0.001	0.154	−0.003	0.128	−0.0003	0.127
UQB		0.067		0.004		0.065		0.002		0.006		0.004	
120	*β*_1_	0.001	0.046	0.0003	0.046	0.001	0.049	<0.0001	0.049	0.0002	0.052	−0.0002	0.052
	*β*_2_	−0.001	0.075	0.0003	0.075	0.001	0.077	−0.0002	0.078	0.006	0.081	0.002	0.081
	*β*_3_	−0.0003	0.077	−0.001	0.077	−0.0003	0.081	−0.001	0.081	0.001	0.089	0.001	0.089
	*β*_4_	−0.0002	0.065	−0.0002	0.065	−0.001	0.066	−0.008	0.066	−0.0003	0.070	0.0002	0.071
	*γ*_1_	0.043	0.134	<0.000	0.128	0.043	0.135	0.001	0.128	0.039	0.133	<0.0001	0.127
	*γ*_2_	−0.001	0.165	0.0004	0.164	−0.001	0.123	<0.0001	0.124	−0.003	0.105	−0.001	0.104
UQB		0.043		0.001		0.043		0.008		0.004		0.002	

**Table 2:** Relative biases and root mean square errors of the Maximum Likelihood Estimators (MLEs) and bootstrap corrected MLEs of the model parameters: $\text{Logit}\left(\mu_{t}/1-\mu_{t}\right)=\beta_{1}+x_{t2}^{\beta_{2}}+\beta_{3}x_{t3}+\beta_{4}x_{t4}$ and $\text{log}\left(\sigma_{t}^{2}\right)=\gamma_{1}+z_{t}^{\gamma_{2}}$, *β* = (−1.7, −1.8, 1.2, −1.3)^⊤^, *μ*_*t*_ ∈ (0.19, 0.86), *t* = 1, …, *n*.

*μ*_*t*_ ∈ (0.19, 0.86)													
*n*	*θ*	λ ≈ 12				λ ≈ 45				λ ≈ 128			
		MLE		BOOT		MLE		BOOT		MLE		BOOT	
		Bias	$\sqrt{MSE}$	Bias	$\sqrt{MSE}$	Bias	$\sqrt{MSE}$	Bias	$\sqrt{MSE}$	Bias	$\sqrt{MSE}$	Bias	$\sqrt{MSE}$
40	*β*_1_	0.002	0.119	0.001	0.119	0.002	0.125	<0.0001	0.124	0.003	0.126	0.001	0.126
	*β*_2_	0.001	0.098	−0.0004	0.097	0.001	0.102	<0.0001	0.101	0.001	0.102	−0.001	0.101
	*β*_3_	0.007	0.220	0.004	0.219	0.004	0.232	0.001	0.232	0.007	0.237	0.003	0.236
	*β*_4_	0.005	0.177	0.001	0.177	0.008	0.176	0.003	0.175	0.004	0.180	−0.0004	0.180
	*γ*_1_	0.131	0.274	−0.004	0.241	0.139	0.275	0.001	0.236	0.137	0.273	−0.001	0.235
	*γ*_2_	−0.020	0.333	0.0003	0.309	0.002	0.235	0.003	0.225	0.004	0.193	0.001	0.188
UQB		0.133		0.006		0.139		0.004		0.137		0.004	
80	*β*_1_	0.001	0.083	0.0002	0.083	0.001	0.087	<0.0001	0.086	0.001	0.088	−0.0003	0.088
	*β*_2_	0.001	0.068	−0.0004	0.068	0.0003	0.071	−0.001	0.071	0.001	0.072	<0.0001	0.071
	*β*_3_	0.001	0.154	−0.001	0.154	0.002	0.162	0.0003	0.162	0.002	0.166	0.0003	0.166
	*β*_4_	0.002	0.124	0.0002	0.123	0.003	0.127	0.0004	0.126	0.002	0.127	0.0002	0.127
	*γ*_1_	0.064	0.171	<0.0001	0.159	0.065	0.171	<0.0001	0.158	0.066	0.170	<0.0001	0.157
	*γ*_2_	−0.005	0.209	0.003	0.202	0.0004	0.154	0.001	0.152	0.002	0.127	0.001	0.126
UQB		0.064		0.003		0.065		0.002		0.066		0.001	
120	*β*_1_	0.001	0.068	<0.0001	0.068	0.001	0.070	<0.0001	0.070	0.0003	0.072	−0.001	0.072
	*β*_2_	0.001	0.055	<0.0001	0.055	0.001	0.058	<0.0001	0.058	0.001	0.058	−0.0002	0.058
	*β*_3_	0.001	0.126	−0.001	0.126	0.0002	0.132	−0.001	0.132	0.001	0.134	<0.0001	0.134
	*β*_4_	0.002	0.100	0.001	0.100	0.002	0.102	0.001	0.102	0.001	0.104	−0.0003	0.104
	*γ*_1_	0.042	0.134	<0.0001	0.127	0.043	0.134	<0.0001	0.127	0.043	0.134	0.0003	0.127
	*γ*_2_	−0.004	0.168	0.0004	0.165	−0.0002	0.124	<0.0001	0.123	0.001	0.104	0.0003	0.104
UQB		0.042		0.001		0.043		0.001		0.043		0.001	

**Table 3:** Relative biases and root mean square errors of the Maximum Likelihood Estimators (MLEs-asymptotic) and bootstrap corrected MLEs of the model parameters:$\text{Logit}\left(\mu_{t}/1-\mu_{t}\right)=\beta_{1}+x_{t2}^{\beta_{2}}+\beta_{3}x_{t3}+\beta_{4}x_{t4}$ and $\text{log}\left(\sigma_{t}^{2}\right)=\gamma_{1}+z_{t}^{\gamma_{2}}$, *β* = (2.1, −1.5, −1.6, −1.2)^⊤^, *μ*_*t*_ ∈ (0.78, 0.98), *t* = 1, …, *n*.

*μ*_*t*_ ∈ (0.78, 0.98)													
*n*	*θ*	λ ≈ 12				λ ≈ 45				λ ≈ 128			
		MLE		BOOT		MLE		BOOT		MLE		BOOT	
		Bias	$\sqrt{MSE}$	Bias	$\sqrt{MSE}$	Bias	$\sqrt{MSE}$	Bias	$\sqrt{MSE}$	Bias	$\sqrt{MSE}$	Bias	$\sqrt{MSE}$
40	*β*_1_	0.001	0.063	0.0003	0.063	0.001	0.067	<0.0001	0.067	0.001	0.067	<0.0001	0.067
	*β*_2_	−0.0003	0.056	<0.0001	0.056	−0.0003	0.059	<0.0001	0.059	−0.001	0.062	−0.0002	0.061
	*β*_3_	0.0004	0.123	0.001	0.123	<0.0001	0.129	0.0003	0.129	−0.001	0.132	−0.001	0.132
	*β*_4_	−0.002	0.097	−0.001	0.097	0.0004	0.101	0.001	0.101	0.0003	0.101	0.001	0.101
	*γ*_1_	0.130	0.271	−0.004	0.238	0.137	0.273	−0.001	0.236	0.138	0.274	−0.001	0.237
	*γ*_2_	−0.023	0.336	0.002	0.310	−0.0004	0.239	−0.0001	0.228	0.004	0.191	0.0004	0.186
UQB		0.132		0.005		0.137		0.001		0.138		0.002	
80	*β*_1_	0.0004	0.044	<0.0001	0.044	0.0004	0.047	<0.0001	0.047	0.001	0.048	0.0004	0.048
	*β*_2_	<0.0001	0.039	<0.0001	0.039	−0.001	0.041	−0.0004	0.041	−0.001	0.043	−0.001	0.043
	*β*_3_	0.001	0.086	0.001	0.086	−0.001	0.091	−0.001	0.091	−0.0003	0.093	−0.0002	0.093
	*β*_4_	0.001	0.069	0.001	0.070	0.0004	0.071	0.001	0.071	<0.0001	0.072	0.001	0.072
	*γ*_1_	0.064	0.171	0.001	0.159	0.067	0.172	0.001	0.158	0.067	0.172	0.000	0.159
	*γ*_2_	−0.008	0.215	0.001	0.207	0.001	0.156	0.001	0.153	0.003	0.129	0.001	0.128
UQB		0.065		0.002		0.067		0.002		0.067		0.002	
120	*β*_1_	0.0002	0.037	<0.0001	0.037	0.001	0.038	0.0002	0.038	0.0003	0.039	<0.0001	0.039
	*β*_2_	0.0003	0.031	0.0004	0.031	<0.0001	0.034	<0.0001	0.034	<0.0001	0.035	<0.0001	0.035
	*β*_3_	<0.0001	0.071	<0.0001	0.071	0.0003	0.075	0.0004	0.075	<0.0001	0.075	<0.0001	0.075
	*β*_4_	−0.001	0.056	−0.001	0.056	−0.001	0.057	−0.0004	0.057	−0.0003	0.059	<0.0001	0.059
	*γ*_1_	0.042	0.134	0.001	0.128	0.046	0.136	0.003	0.128	0.045	0.134	0.001	0.128
	*γ*_2_	−0.004	0.168	0.001	0.164	0.001	0.123	0.001	0.121	0.002	0.102	0.001	0.102
UQB		0.042		0.002		0.046		0.003		0.045		0.001	

For example, the relative bias estimate of the ${\bar{\beta }}_{3}$ (BOOT) estimator is equal to 0.0003, while that of the ${\hat{\beta }}_{3}$ (MLE-asymptotic) is 0.001. For *μ*_*t*_ ∈ (0.19, 0.86), *n* = 120 and λ ≈ 12, the estimated biased is equal to 0.001 for ${\hat{\beta }}_{2}$ and < 0.0001 for ${\bar{\beta }}_{2}$. In fact, it is noteworthy the high performance of the bootstrap correction when *μ*_*t*_ ∈ (0.19, 0.86), since its estimators exhibit lower biases than the MLEs-asymptotic for all model parameters, for the different levels of non-constant dispersion and the sample sizes. In all scenarios considered, we note that the RMSEs of the estimators decrease when the sample size increases.

As we had expected the MLEs-asymptotic of the parameters of the dispersion submodel tend to be more biased than those of the mean submodel, especially regarding *γ*_1_. For instance, for *μ*_*t*_ ∈ (0.02, 0.32), *n* = 120 and λ ≈ 45, the relative bias estimate of ${\hat{\gamma }}_{1}$ is equal to 0.043, while that of the ${\bar{\gamma }}_{1}$ is < 0.0001. More expressive are the biases of ${\hat{\gamma }}_{1}$ and ${\hat{\gamma }}_{2}$ which drop from (0.136, −0.010) to (−0.001,0.001) after the bootstrap correction, respectively, when *n* = 40 and λ ≈ 45.

For ${\hat{\gamma }}_{1}$ in particular, the bootstrap correction provides a substantial reduction of the estimated bias. This is important since the correct estimation of the of the dispersion submodel parameters, directly interferes with the estimates of the response variances, which, when corrected, produce Z-tests that lead to truer decisions. Even so, the corrections were also effective for ${\hat{\beta }}_{i}$, *i* = 1, 2, 3, 4 because the goal is for the bias values to be as close to zero as possible, and for these parameters, through correction, the estimated bias became some times < 0.0001, i.e. the goal was achieved.

It is important to note that the estimated biases of the usual and corrected maximum likelihood estimators are notably smaller when the mean of the response variable is close to the upper limit of the unit interval than for the two other scenarios considered (Tables 1 and 3). Based on the Unified Quadratic Bias measure it becomes more evident how effective the bias correction we propose is. Let shall evaluated the results on *μ*_*t*_ ∈ (0.78, 0.98) and *n* = 40, for λ ≈ 12, 45 and 128, we have that the values of the UQB for the original MLEs are equal to 0.132, 0.137 and 0.138, whereas for the corrected version these values became 0.005, 0.001 and 0.002, respectively (Table 3).

## 6 Numerical results on confidence intervals

Concerning interval estimation, we computing the empirical coverage of the intervals (%), obtained from the relative frequencies in which the intervals contained the true value of the parameter. The lower and upper bounds were also estimated (via the average after the end of the Monte Carlo process), thus we were able to estimate the average length of the intervals and left and right non-coverage rates. The left rate is computed whenever the interval upper limit is less than the true value of the parameter and right rate is computed whenever the interval lower limit is greater than true value of the parameter.

In what follows we report the results of Monte Carlo simulations on interval estimation. We shall just take the nominal levels 0.90 and 0.95 concerning to Tables 4 and 5, respectively. These tables display the coverage rates of the following competing interval estimators: the asymptotic ML-like or ML interval approximation (ML-I_*a*_), bootstrap_*t*_ (Boot_*t*_) and percentile (Boot*p*) for the model parameters in (6).

**Table 4:** Coverage rates of the interval estimators: ML-I_*a*_, Boot_*t*_ and Boot*p* for *θ*, the model parameters: $\text{Logit}\left(\mu_{t}/1-\mu_{t}\right)=\beta_{1}+x_{t2}^{\beta_{2}}+\beta_{3}x_{t3}+\beta_{4}x_{t4}$ and $\text{log}\left(\sigma_{t}^{2}\right)=\gamma_{1}+z_{t}^{\gamma_{2}}$, *t* = 1, …, *n*, 1 − *α* = 0.90.

*μ*_*t*_ ∈ (0.02, 0.32)										
n	*θ*	λ ≈ 12			λ ≈ 45			λ ≈ 128		
		ML-I_*a*_	Boot_*t*_	Boot*p*	ML-I_*a*_	Boot_*t*_	Boot*p*	ML-I_*a*_	Boot_*t*_	Boot*p*
40	*β*_1_	0.852	0.898	0.858	0.853	0.897	0.858	0.859	0.897	0.862
	*β*_2_	0.859	0.902	0.868	0.858	0.896	0.862	0.856	0.899	0.855
	*β*_3_	0.848	0.893	0.855	0.849	0.894	0.855	0.862	0.900	0.864
	*β*_4_	0.853	0.892	0.857	0.853	0.892	0.856	0.857	0.896	0.859
	*γ*_1_	0.800	0.894	0.663	0.804	0.902	0.663	0.808	0.905	0.658
	*γ*_2_	0.853	0.894	0.889	0.861	0.891	0.902	0.863	0.892	0.897
80	*β*_1_	0.881	0.900	0.881	0.880	0.899	0.885	0.882	0.899	0.881
	*β*_2_	0.886	0.901	0.886	0.882	0.895	0.885	0.884	0.903	0.881
	*β*_3_	0.883	0.902	0.885	0.883	0.901	0.884	0.883	0.900	0.884
	*β*_4_	0.880	0.895	0.880	0.881	0.898	0.882	0.882	0.897	0.881
	*γ*_1_	0.852	0.896	0.768	0.847	0.892	0.770	0.861	0.897	0.791
	*γ*_2_	0.879	0.889	0.899	0.881	0.887	0.902	0.884	0.893	0.897
120	*β*_1_	0.883	0.898	0.883	0.876	0.886	0.876	0.887	0.898	0.887
	*β*_2_	0.890	0.900	0.889	0.900	0.896	0.892	0.888	0.900	0.885
	*β*_3_	0.886	0.896	0.884	0.877	0.889	0.877	0.886	0.898	0.886
	*β*_4_	0.885	0.897	0.885	0.890	0.900	0.888	0.889	0.899	0.890
	*γ*_1_	0.872	0.898	0.815	0.868	0.896	0.815	0.875	0.900	0.827
	*γ*_2_	0.885	0.893	0.899	0.891	0.893	0.904	0.884	0.890	0.892
*μ*_*t*_ ∈ (0.19, 0.86)										
n	*θ*	λ ≈ 12			λ ≈ 45			λ ≈ 128		
		ML-I_*a*_	Boot_*t*_	Boot*p*	ML-I_*a*_	Boot_*t*_	Boot*p*	ML-I_*a*_	Boot_*t*_	Boot*p*
40	*β*_1_	0.854	0.899	0.862	0.853	0.898	0.859	0.860	0.902	0.862
	*β*_2_	0.851	0.902	0.856	0.853	0.900	0.855	0.861	0.906	0.865
	*β*_3_	0.855	0.898	0.862	0.853	0.898	0.859	0.858	0.903	0.861
	*β*_4_	0.859	0.900	0.862	0.860	0.901	0.863	0.861	0.899	0.864
	*γ*_1_	0.803	0.896	0.673	0.801	0.902	0.660	0.803	0.904	0.658
	*γ*_2_	0.848	0.906	0.886	0.853	0.895	0.891	0.861	0.897	0.898
80	*β*_1_	0.877	0.896	0.879	0.880	0.901	0.880	0.879	0.899	0.880
	*β*_2_	0.873	0.898	0.876	0.876	0.900	0.875	0.883	0.904	0.884
	*β*_3_	0.875	0.896	0.877	0.879	0.898	0.878	0.881	0.901	0.883
	*β*_4_	0.880	0.898	0.879	0.879	0.898	0.878	0.884	0.902	0.881
	*γ*_1_	0.858	0.903	0.781	0.855	0.902	0.775	0.856	0.904	0.776
	*γ*_2_	0.879	0.898	0.899	0.882	0.900	0.900	0.884	0.901	0.903
120	*β*_1_	0.885	0.900	0.885	0.886	0.900	0.889	0.885	0.898	0.886
	*β*_2_	0.880	0.899	0.882	0.884	0.897	0.883	0.889	0.904	0.900
	*β*_3_	0.885	0.898	0.887	0.886	0.897	0.885	0.886	0.898	0.886
	*β*_4_	0.888	0.899	0.888	0.891	0.903	0.900	0.889	0.902	0.900
	*γ*_1_	0.873	0.904	0.820	0.870	0.900	0.816	0.873	0.903	0.811
	*γ*_2_	0.885	0.895	0.894	0.885	0.895	0.897	0.887	0.897	0.898
*μ*_*t*_ ∈ (0.78, 0.98)										
n	*θ*	λ ≈ 12			λ ≈ 45			λ ≈ 128		
		ML-I_*a*_	Boot_*t*_	Boot*p*	ML-I_*a*_	Boot_*t*_	Boot*p*	ML-I_*a*_	Boot_*t*_	Boot*p*
40	*β*_1_	0.857	0.900	0.863	0.853	0.894	0.855	0.858	0.898	0.860
	*β*_2_	0.848	0.897	0.858	0.851	0.897	0.859	0.859	0.901	0.862
	*β*_3_	0.859	0.898	0.864	0.859	0.902	0.863	0.858	0.896	0.861
	*β*_4_	0.860	0.900	0.866	0.856	0.897	0.858	0.863	0.900	0.866
	*γ*_1_	0.814	0.898	0.681	0.804	0.901	0.664	0.804	0.897	0.657
	*γ*_2_	0.846	0.906	0.886	0.851	0.894	0.887	0.859	0.899	0.894
80	*β*_1_	0.882	0.902	0.882	0.876	0.897	0.875	0.880	0.898	0.878
	*β*_2_	0.878	0.897	0.881	0.879	0.898	0.882	0.880	0.899	0.880
	*β*_3_	0.877	0.894	0.879	0.877	0.894	0.878	0.880	0.897	0.880
	*β*_4_	0.877	0.895	0.877	0.881	0.897	0.880	0.875	0.890	0.875
	*γ*_1_	0.850	0.899	0.779	0.850	0.903	0.770	0.851	0.899	0.769
	*γ*_2_	0.871	0.890	0.890	0.879	0.899	0.898	0.881	0.897	0.898
120	*β*_1_	0.882	0.895	0.883	0.884	0.895	0.885	0.891	0.901	0.887
	*β*_2_	0.890	0.901	0.892	0.893	0.903	0.892	0.885	0.895	0.885
	*β*_3_	0.886	0.896	0.885	0.879	0.890	0.879	0.887	0.899	0.886
	*β*_4_	0.884	0.896	0.884	0.887	0.899	0.886	0.884	0.898	0.884
	*γ*_1_	0.871	0.904	0.816	0.871	0.900	0.808	0.865	0.900	0.807
	*γ*_2_	0.888	0.902	0.902	0.887	0.901	0.898	0.883	0.895	0.894

**Table 5:** Coverage rates of the interval estimators: ML-I_*a*_, Boot_*t*_ and Boot*p* for *θ*, the model parameters: $\text{Logit}\left(\mu_{t}/1-\mu_{t}\right)=\beta_{1}+x_{t2}^{\beta_{2}}+\beta_{3}x_{t3}+\beta_{4}x_{t4}$ and $\text{log}\left(\sigma_{t}^{2}\right)=\gamma_{1}+z_{t}^{\gamma_{2}}$, *t* = 1, …, *n*, 1 − *α* = 0.95.

*μ*_*t*_ ∈ (0.02, 0.32)										
n	*θ*	λ ≈ 12			λ ≈ 45			λ ≈ 128		
		ML-I_*a*_	Boot_*t*_	Boot*p*	ML-I_*a*_	Boot_*t*_	Boot*p*	ML-I_*a*_	Boot_*t*_	Boot*p*
40	*β*_1_	0.913	0.948	0.919	0.915	0.949	0.918	0.918	0.949	0.920
	*β*_2_	0.919	0.950	0.923	0.919	0.950	0.922	0.917	0.949	0.916
	*β*_3_	0.909	0.944	0.914	0.914	0.947	0.916	0.917	0.948	0.918
	*β*_4_	0.911	0.938	0.912	0.914	0.942	0.914	0.918	0.945	0.916
	*γ*_1_	0.872	0.945	0.744	0.872	0.951	0.744	0.876	0.951	0.745
	*γ*_2_	0.915	0.954	0.944	0.921	0.939	0.950	0.923	0.940	0.948
80	*β*_1_	0.934	0.949	0.934	0.933	0.948	0.934	0.937	0.951	0.937
	*β*_2_	0.937	0.951	0.938	0.935	0.946	0.937	0.935	0.952	0.934
	*β*_3_	0.936	0.951	0.937	0.936	0.951	0.937	0.937	0.948	0.937
	*β*_4_	0.933	0.947	0.934	0.935	0.944	0.933	0.937	0.948	0.936
	*γ*_1_	0.914	0.948	0.840	0.907	0.944	0.838	0.917	0.947	0.860
	*γ*_2_	0.936	0.942	0.950	0.937	0.940	0.948	0.941	0.942	0.946
120	*β*_1_	0.939	0.946	0.937	0.931	0.941	0.932	0.942	0.950	0.941
	*β*_2_	0.941	0.948	0.941	0.942	0.946	0.942	0.940	0.951	0.939
	*β*_3_	0.938	0.947	0.936	0.934	0.944	0.934	0.942	0.950	0.941
	*β*_4_	0.941	0.949	0.940	0.942	0.947	0.939	0.941	0.947	0.941
	*γ*_1_	0.928	0.946	0.879	0.923	0.948	0.878	0.931	0.950	0.889
	*γ*_2_	0.939	0.943	0.948	0.942	0.944	0.950	0.940	0.942	0.946
*μ*_*t*_ ∈ (0.19, 0.86)										
n	*θ*	λ ≈ 12			λ ≈ 45			λ ≈ 128		
		ML-I_*a*_	Boot_*t*_	Boot*p*	ML-I_*a*_	Boot_*t*_	Boot*p*	ML-I_*a*_	Boot_*t*_	Boot*p*
40	*β*_1_	0.913	0.948	0.918	0.914	0.947	0.917	0.916	0.950	0.917
	*β*_2_	0.909	0.950	0.915	0.914	0.951	0.918	0.921	0.951	0.923
	*β*_3_	0.914	0.951	0.918	0.913	0.948	0.914	0.918	0.951	0.920
	*β*_4_	0.915	0.946	0.919	0.919	0.953	0.921	0.917	0.950	0.920
	*γ*_1_	0.872	0.946	0.754	0.867	0.951	0.743	0.871	0.949	0.740
	*γ*_2_	0.915	0.964	0.943	0.915	0.943	0.946	0.920	0.943	0.948
80	*β*_1_	0.934	0.950	0.934	0.934	0.950	0.935	0.933	0.950	0.935
	*β*_2_	0.930	0.948	0.932	0.932	0.948	0.932	0.936	0.952	0.937
	*β*_3_	0.930	0.948	0.931	0.930	0.945	0.933	0.937	0.950	0.936
	*β*_4_	0.934	0.948	0.934	0.933	0.948	0.933	0.937	0.950	0.935
	*γ*_1_	0.917	0.948	0.847	0.914	0.948	0.845	0.916	0.950	0.846
	*γ*_2_	0.937	0.947	0.949	0.939	0.946	0.949	0.942	0.949	0.953
120	*β*_1_	0.940	0.949	0.942	0.940	0.947	0.939	0.941	0.949	0.942
	*β*_2_	0.937	0.947	0.938	0.937	0.947	0.937	0.939	0.950	0.939
	*β*_3_	0.940	0.948	0.938	0.939	0.950	0.937	0.939	0.947	0.939
	*β*_4_	0.941	0.950	0.941	0.941	0.950	0.941	0.943	0.952	0.942
	*γ*_1_	0.927	0.951	0.882	0.927	0.950	0.881	0.930	0.948	0.880
	*γ*_2_	0.937	0.942	0.946	0.939	0.945	0.947	0.940	0.946	0.949
*μ*_*t*_ ∈ (0.78, 0.98)										
n	*θ*	λ ≈ 12			λ ≈ 45			λ ≈ 128		
		ML-I_*a*_	Boot_*t*_	Boot*p*	ML-I_*a*_	Boot_*t*_	Boot*p*	ML-I_*a*_	Boot_*t*_	Boot*p*
40	*β*_1_	0.915	0.951	0.921	0.913	0.946	0.916	0.917	0.948	0.918
	*β*_2_	0.907	0.945	0.916	0.912	0.947	0.919	0.917	0.947	0.918
	*β*_3_	0.918	0.950	0.921	0.921	0.950	0.923	0.914	0.944	0.917
	*β*_4_	0.917	0.948	0.920	0.918	0.949	0.920	0.921	0.950	0.920
	*γ*_1_	0.873	0.946	0.759	0.873	0.949	0.749	0.868	0.949	0.743
	*γ*_2_	0.913	0.961	0.942	0.915	0.941	0.945	0.917	0.945	0.948
80	*β*_1_	0.938	0.950	0.937	0.933	0.946	0.932	0.932	0.946	0.933
	*β*_2_	0.933	0.950	0.936	0.934	0.949	0.936	0.935	0.947	0.934
	*β*_3_	0.935	0.949	0.936	0.934	0.948	0.935	0.938	0.948	0.936
	*β*_4_	0.931	0.943	0.932	0.932	0.945	0.933	0.930	0.943	0.929
	*γ*_1_	0.912	0.947	0.847	0.914	0.950	0.841	0.915	0.949	0.841
	*γ*_2_	0.932	0.943	0.943	0.936	0.946	0.949	0.934	0.944	0.946
120	*β*_1_	0.937	0.947	0.938	0.937	0.947	0.936	0.941	0.949	0.943
	*β*_2_	0.942	0.951	0.942	0.941	0.950	0.942	0.940	0.948	0.940
	*β*_3_	0.936	0.943	0.937	0.933	0.942	0.934	0.938	0.947	0.938
	*β*_4_	0.938	0.945	0.939	0.941	0.948	0.939	0.941	0.949	0.939
	*γ*_1_	0.927	0.951	0.881	0.927	0.950	0.875	0.925	0.947	0.872
	*γ*_2_	0.940	0.948	0.949	0.940	0.947	0.949	0.938	0.945	0.947

Regarding coverage rates the interval that performs best is the bootstrap_*t*_, with empirical coverage substantially closest to the nominal levels, for all parameters model. The asymptotic confidence interval displayed considerable undercoverage and the percentile confidence interval Boot*p* overall outperforms the ML-I_*a*_, only for *γ*_1_ the Boot*p* displays a poor performance. For instance, consider *n* = 40, *μ*_*t*_ ∈ (0.19, 0.86), 1 − *α* = 0.90 and all λ′ *s* values, the Boot*p* coverage ratios for this parameter are approximately equal to 0.66. Whereas those of the ML-I_*a*_ and Boot_*t*_ are approximately equal to 0.80 and 0.90, respectively. These behavior are similar for all scenarios of the mean response. The conclusions about coverage rates are quite similar whatever the nominal level is. To exemplify, for 1 − *α* = 0.95 and for the same other settings when 1 − *α* = 0.90, the ML-I_*a*_, Boot_*t*_ and Boot*p* coverage rates are around 0.87, 0.95 and 0.74, respectively (Table 5). Now consider *n* = 120, 1 − *α* = 0.95, λ = 45, *μ*_*t*_ ∈ (0.19, 0.86), as to *β*_3_, those values are equal to 0.933, 0.942 and 0.934 (Table 8). Meaning, even when the size of the sample increases the empirical coverage of the Boot_*t*_ interval is closest to the nominal level.

Our interest hereafter shall lie in evaluate some interval properties only for the nonlinearity parameters of the mean and the dispersion submodels, meaning *β*_2_ and *γ*_2_. Tables 6 through 9 present the mean lower (Lower) and upper (Upper) bounds, mean lengths (Size), the empirical probability of coverage (Coverage), as well as the left and right coverage rates (%) of the interval estimators of the previously mentioned parameters. These last two quantities evaluate the balancing of the interval. Perfect balancing occurs when these two percentages (%) are identical.

**Table 6:** Lower and upper bounds, size, empirical coverage (Coverage) and percentages of Lower (%Left) and upper (%Right) non-coverage of the ML-I_*a*_, Boot_*t*_ and Boot*p* intervals for *β*_2_, in the model: $\text{Logit}\left(\mu_{t}/1-\mu_{t}\right)=\beta_{1}+x_{t2}^{\beta_{2}}+\beta_{3}x_{t3}+\beta_{4}x_{t4}$ and $\text{log}\left(\sigma_{t}^{2}\right)=\gamma_{1}+z_{t}^{\gamma_{2}}$, *t* = 1, …, *n*, *μ*_*t*_ ∈ (0.02, 0.32), *β*_2_ = 1.2, *n* = 40.

λ ≈ 12							
*α*	Intervals	Lower	Upper	Size	Coverage	%Left	%Right
10%	ML-I_*a*_	1.153	1.644	0.490	0.859	6.61	7.46
	Boot_*t*_	1.127	1.683	0.556	0.902	4.74	5.10
	Boot*p*	1.147	1.644	0.497	0.868	5.97	7.26
5%	ML-I_*a*_	1.107	1.691	0.584	0.919	3.61	4.49
	Boot_*t*_	1.074	1.744	0.670	0.950	2.40	2.61
	Boot*p*	1.097	1.689	0.592	0.923	3.20	4.47
1%	ML-I_*a*_	1.015	1.783	0.768	0.976	0.83	1.60
	Boot_*t*_	0.964	1.869	0.905	0.991	0.34	0.57
	Boot*p*	1.002	1.777	0.775	0.976	0.83	1.56
λ ≈ 45							
*α*	Intervals	Lower	Upper	Size	Coverage	%Left	%Right
10%	ML-I_*a*_	1.154	1.657	0.503	0.858	7.23	6.95
	Boot_*t*_	1.127	1.692	0.565	0.896	5.63	4.75
	Boot*p*	1.152	1.657	0.506	0.862	7.00	7.30
5%	ML-I_*a*_	1.106	1.705	0.599	0.919	4.10	5.03
	Boot_*t*_	1.074	1.755	0.681	0.950	2.78	2.24
	Boot*p*	1.101	1.702	0.602	0.922	3.77	4.07
1%	ML-I_*a*_	1.012	1.799	0.788	0.977	1.10	1.23
	Boot_*t*_	0.962	1.883	0.922	0.988	0.70	0.50
	Boot*p*	1.004	1.792	0.789	0.977	1.01	1.33
λ ≈ 128							
*α*	Intervals	Lower	Upper	Size	Coverage	%Left	%Right
10%	ML-I_*a*_	0.938	1.471	0.533	0.856	7.01	7.41
	Boot_*t*_	0.907	1.508	0.600	0.899	5.21	4.92
	Boot*p*	0.937	1.474	0.538	0.855	6.20	7.25
5%	ML-I_*a*_	0.887	1.522	0.635	0.917	3.94	4.40
	Boot_*t*_	0.850	1.575	0.725	0.949	2.66	2.39
	Boot*p*	0.883	1.522	0.639	0.916	3.98	4.43
1%	ML-I_*a*_	0.787	1.621	0.834	0.973	1.10	1.58
	Boot_*t*_	0.731	1.712	0.981	0.988	0.61	0.54
	Boot*p*	0.781	1.617	0.836	0.973	1.09	1.58

In Tables 6–8 are presented the results for *β*_2_, *n* = 40, *n* = 80 and *n* = 120, and only for *μ*_*t*_ ∈ (0.02, 0.32), *t* = 1…, *n*, where *β*_2_ = 1.2. Note that if *β*_2_ = 1 the mean submodel becomes linear. For *n* = 40, λ ≈ 12 and λ ≈ 45 only the Boot_*t*_ interval and for 1− *α* = 0.99 considers this possibility.

**Table 7:** Lower and upper bounds, size, empirical coverage (Coverage) and percentages of Lower (%Left) and upper (%Right) non-coverage of the ML-I_*a*_, Boot_*t*_ and Boot*p* intervals for *β*_2_, in the model: $\text{Logit}\left(\mu_{t}/1-\mu_{t}\right)=\beta_{1}+x_{t2}^{\beta_{2}}+\beta_{3}x_{t3}+\beta_{4}x_{t4}$ and $\text{log}\left(\sigma_{t}^{2}\right)=\gamma_{1}+z_{t}^{\gamma_{2}}$, *t* = 1, …, *n*, *μ*_*t*_ ∈ (0.02, 0.32), *β*_2_ = 1.2, *n* = 80.

λ ≈ 12							
*α*	Intervals	Lower	Upper	Size	Coverage	%Left	%Right
10%	ML-I_*a*_	1.218	1.579	0.362	0.886	5.39	6.03
	Boot_*t*_	1.210	1.593	0.383	0.901	4.79	5.10
	Boot*p*	1.215	1.579	0.363	0.886	5.24	6.11
5%	ML-I_*a*_	1.183	1.614	0.431	0.937	2.78	3.47
	Boot_*t*_	1.175	1.633	0.459	0.951	2.35	2.56
	Boot*p*	1.179	1.612	0.433	0.938	2.61	3.63
1%	ML-I_*a*_	1.115	1.682	0.567	0.985	0.54	0.93
	Boot_*t*_	1.103	1.713	0.610	0.988	0.53	0.62
	Boot*p*	1.110	1.677	0.567	0.984	0.55	1.07
λ ≈ 45							
*α*	Intervals	Lower	Upper	Size	Coverage	%Left	%Right
10%	ML-I_*a*_	1.217	1.588	0.371	0.882	5.98	5.79
	Boot_*t*_	1.208	1.597	0.390	0.895	5.30	5.18
	Boot*p*	1.218	1.588	0.370	0.885	5.99	5.55
5%	ML-I_*a*_	1.181	1.623	0.442	0.935	3.19	3.35
	Boot_*t*_	1.172	1.639	0.467	0.946	2.80	2.63
	Boot*p*	1.181	1.622	0.441	0.937	2.97	3.29
1%	ML-I_*a*_	1.112	1.693	0.581	0.983	0.76	0.95
	Boot_*t*_	1.098	1.720	0.621	0.988	0.62	0.59
	Boot*p*	1.110	1.688	0.578	0.981	0.80	1.08
λ ≈ 128							
*α*	Intervals	Lower	Upper	Size	Coverage	%Left	%Right
10%	ML-I_*a*_	1.006	1.401	0.395	0.884	5.93	5.69
	Boot_*t*_	0.993	1.410	0.417	0.903	4.92	4.74
	Boot*p*	1.010	1.406	0.396	0.881	6.53	5.32
5%	ML-I_*a*_	0.969	1.439	0.470	0.935	3.29	3.22
	Boot_*t*_	0.955	1.454	0.500	0.952	2.60	2.17
	Boot*p*	0.970	1.442	0.472	0.934	3.49	3.15
1%	ML-I_*a*_	0.895	1.513	0.618	0.983	0.79	0.94
	Boot_*t*_	0.876	1.542	0.665	0.988	0.63	0.54
	Boot*p*	0.895	1.513	0.618	0.981	0.90	1.02

**Table 8:** Lower and upper bounds, size, empirical coverage (Coverage) and percentages of lower (%Left) and upper (%Right) non-coverage of the ML-I_*a*_, Boot_*t*_ and Boot*p* intervals for *β*_2_, in the model: $\text{Logit}\left(\mu_{t}/1-\mu_{t}\right)=\beta_{1}+x_{t2}^{\beta_{2}}+\beta_{3}x_{t3}+\beta_{4}x_{t4}$ and $\text{log}\left(\sigma_{t}^{2}\right)=\gamma_{1}+z_{t}^{\gamma_{2}}$, *t* = 1, …, *n*, *μ*_*t*_ ∈ (0.02, 0.32), *β*_2_ = 1.2, *n* = 120.

λ ≈ 12							
*α*	Intervals	Lower	Upper	Size	Coverage	%Left	%Right
10%	ML-I_*a*_	1.249	1.549	0.300	0.890	5.21	5.80
	Boot_*t*_	1.246	1.557	0.311	0.900	4.90	5.13
	Boot*p*	1.248	1.549	0.300	0.889	5.18	5.90
5%	ML-I_*a*_	1.221	1.578	0.357	0.941	2.65	3.21
	Boot_*t*_	1.217	1.590	0.372	0.948	2.55	2.67
	Boot*p*	1.218	1.576	0.358	0.940	2.55	3.41
1%	ML-I_*a*_	1.164	1.634	0.469	0.986	0.54	0.83
	Boot_*t*_	1.160	1.652	0.492	0.989	0.51	0.62
	Boot*p*	1.161	1.630	0.469	0.986	0.42	0.99
λ ≈ 45							
*α*	Intervals	Lower	Upper	Size	Coverage	%Left	%Right
10%	ML-I_*a*_	1.247	1.554	0.307	0.890	5.30	5.71
	Boot_*t*_	1.242	1.559	0.316	0.896	4.89	5.55
	Boot*p*	1.249	1.555	0.306	0.892	5.19	5.60
5%	ML-I_*a*_	1.218	1.584	0.366	0.942	2.67	3.11
	Boot_*t*_	1.213	1.592	0.378	0.946	2.64	2.73
	Boot*p*	1.219	1.583	0.364	0.942	2.64	3.18
1%	ML-I_*a*_	1.161	1.641	0.481	0.986	0.56	0.79
	Boot_*t*_	1.155	1.655	0.501	0.988	0.62	0.56
	Boot*p*	1.160	1.638	0.477	0.985	0.58	0.94
λ ≈ 128							
*α*	Intervals	Lower	Upper	Size	Coverage	%Left	%Right
10%	ML-I_*a*_	1.044	1.370	0.326	0.888	5.86	5.37
	Boot_*t*_	1.035	1.372	0.337	0.900	5.13	4.89
	Boot*p*	1.049	1.376	0.327	0.885	6.63	4.83
5%	ML-I_*a*_	1.013	1.402	0.389	0.940	3.19	2.79
	Boot_*t*_	1.004	1.408	0.404	0.951	2.77	2.10
	Boot*p*	1.016	1.405	0.389	0.939	3.57	2.51
1%	ML-I_*a*_	0.952	1.463	0.511	0.986	0.64	0.72
	Boot_*t*_	0.942	1.476	0.534	0.988	0.65	0.51
	Boot*p*	0.954	1.464	0.510	0.984	0.82	0.73

Its empirical probability of coverage is the closest to the true one, however, the its mean length interval is longer than those of the other two intervals, which allows the inclusion of *β*_2_ = 1.0, i.e., linearity (Table 6). This behavior of the Boot_*t*_ interval holds for *n* = 80 (Table 7). When λ ≈ 128 all intervals include the possibility of linearity when *n* = 40, i.e., *β*_2_ = 1.0 (Table 6), for all confidence nominal levels. This result is interesting, as it shows how the intensity of non-constant dispersion negatively affects the performances of the three confidence intervals considered.

As the sample size increases the problem is smoothed. For instance, when *n* = 80, 1 − *α* = 0.90, λ ≈ 128, only the bootstrap_*t*_ interval considers the possibility of linearity, namely: ML-I_*a*_: = (1.006, 1.401), Boot_*t*_: = (0.993, 1.410) and Boot*p*: = (1.010, 1.406). Nevertheless, if we would use only one decimal approximation those intervals would become ML-I_*a*_: = (1.0, 1.4), Boot_*t*_: = (1.0, 1.4) and Boot*p*: = (1.0, 1.4), therefore, admittedly equivalents. We should also point out that the average lengths (Size) of all intervals decrease as the sample size increases.

Our spotlight hereafter is show how the Boot_*t*_ considerably outperforms the accuracy and balance of its competitors, concerning for *β*_2_ interval. We shall fix 1 − *α* = 0.95 and consider *n* = 40 and three λ’s values. We shall compose the following set consisting of: the empirical coverage and left and right non-coverages rates of the interval estimators, expressed as {(⋅), [⋅%][⋅%]}. For λ ≈ 12, the sets of the ML-I_*a*_, Boot_*t*_ and Boot*p* estimators are equal to {(0.919), [3.61%][4.49%]}, {(0.950), [2.40%][2.61%]} and {(0.923), [3.20%][4.47%]}. For λ ≈ 45 those sets have become {(0.919), [2.65%][3.21%]}, {(0.950), [2.55%][2.67%]} and {(0.922), [2.97%][3.41]}. Finally, when λ ≈ 128 the respective sets are {(0.917), [3.94%][4.40%]}, {(0.949), [2.66%][2.39%]} and {(0.916), [3.98%][4.48%]}.

Table 9 present the simulation results for *β*_2_ (*μ*_*t*_ ≈ 0.5 and *μ*_*t*_ ≈ 1), when *n* = 40, λ ≈ 128 and *β*_2_ equal to −1.8 and −1.5, respectively. We note that for the three nominal levels and the different scenarios, the asymptotic type confidence interval has the shortest average length. We also note that the bootstrap_*t*_ confidence interval presents the best empirical coverage and balance proprieties, followed by the Boot*p* interval which had very similar values to the ML-I_*a*_ interval.

**Table 9:** Lower and upper bounds, size, empirical coverage (Coverage) and percentages of lower (%Left) and upper (%Right) non-coverage of the ML-I_*a*_, Boot_*t*_ and Boot*p* intervals. For *β*_2_ in the model: $\text{Logit}\left(\mu_{t}/1-\mu_{t}\right)=\beta_{1}+x_{t2}^{\beta_{2}}+\beta_{3}x_{t3}+\beta_{4}x_{t4}$ and $\text{log}\left(\sigma_{t}^{2}\right)=\gamma_{1}+z_{t}^{\gamma_{2}}$, *t* = 1, …, *n*, *n* = 40, λ ≈ 128.

*μ*_*t*_ ∈ (0.19, 0.86), *β*_2_ = −1.8.							
*α*	Intervals	Lower	Upper	Size	Coverage	%Left	%Right
10%	ML-I_*a*_	−1.994	−1.609	0.385	0.861	5.02	8.83
	Boot_*t*_	−1.998	−1.560	0.439	0.906	4.57	4.80
	Boot*p*	−1.995	−1.603	0.392	0.865	5.15	8.30
5%	ML-I_*a*_	−2.031	−1.573	0.459	0.921	2.46	5.47
	Boot_*t*_	−2.035	−1.505	0.530	0.951	2.27	2.58
	Boot*p*	−2.030	−1.563	0.467	0.923	2.58	5.08
1%	ML-I_*a*_	−2.103	−1.500	0.603	0.971	0.49	2.41
	Boot_*t*_	−2.111	−1.392	0.718	0.989	0.48	0.63
	Boot*p*	−2.095	−1.480	0.615	0.973	0.65	2.03
*μ*_*t*_ ∈ (0.78, 0.98), *β*_2_ = −1.5.							
*α*	Intervals	Lower	Upper	Size	Coverage	%Left	%Right
10%	ML-I_*a*_	−1.614	−1.384	0.230	0.859	6.13	8.00
	Boot_*t*_	−1.624	−1.364	0.261	0.901	4.60	5.32
	Boot*p*	−1.613	−1.379	0.234	0.862	6.39	7.37
5%	ML-I_*a*_	−1.636	−1.362	0.274	0.917	3.27	5.00
	Boot_*t*_	−1.648	−1.334	0.314	0.947	2.47	2.78
	Boot*p*	−1.634	−1.356	0.279	0.918	3.68	4.54
1%	ML-I_*a*_	−1.679	−1.319	0.361	0.972	1.01	1.82
	Boot_*t*_	−1.698	−1.274	0.424	0.987	0.60	0.65
	Boot*p*	−1.675	−1.309	0.366	0.973	1.19	1.55

**Fig 1:**
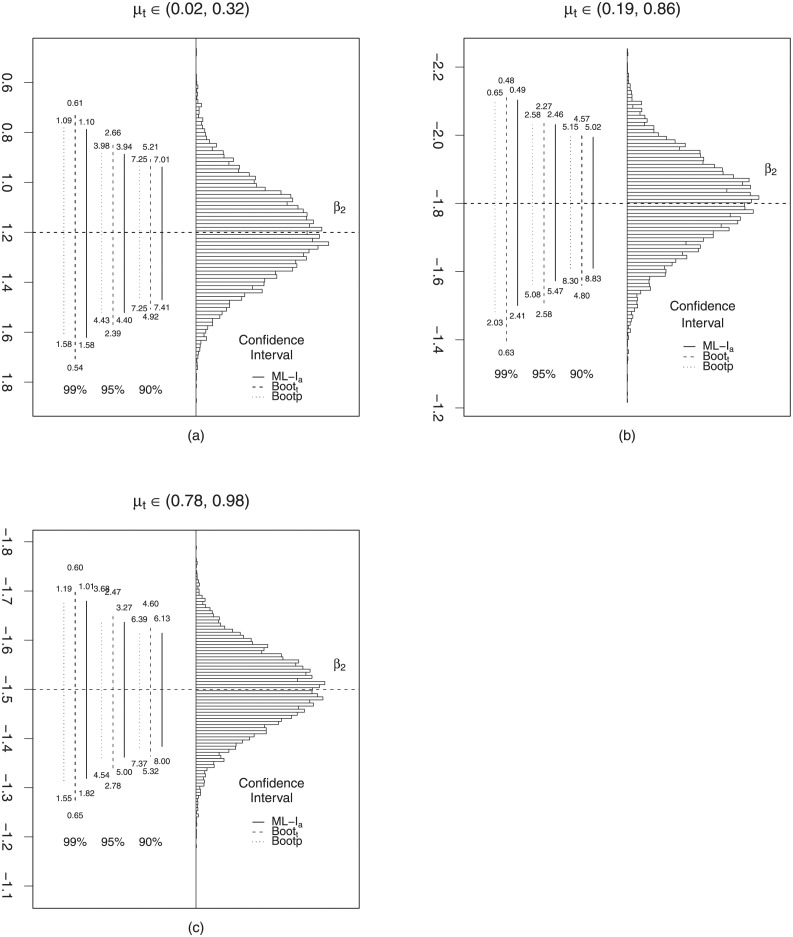
Interval estimation for *β*_2_, *n* = 40, λ ≈ 128. (*a*) *β*_2_ = 1.2, (*b*) *β*_2_ = −1.8 and (*c*) *β*_2_ = −1.5.

**Fig 2:**
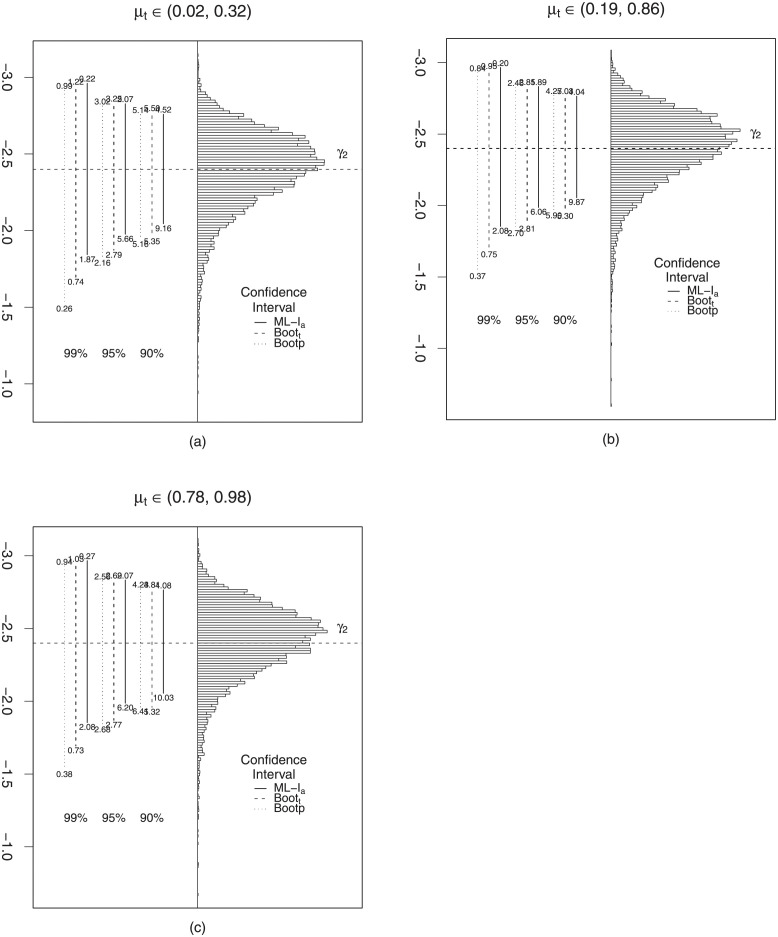
Interval estimation for *γ*_2_, *n* = 40. *γ*_2_ = −2.4.

Figs 1 and 2 contain histograms constructed from the 10000 maximum likelihood estimates of the parameter *β*_2_ and *γ*_2_, respectively, for *n* = 40, λ ≈ 128 and the different scenarios for *μ*_*t*_, *t* = 1, …, *n*. The distinct lines represent the different confidence intervals under evaluation, and their lengths correspond to the respective average lengths. The values below and above of the vertical lines are the non-coverage rates, meaning the percentages of replicates in which the true value of the parameter was smaller than the lower limit of the interval (below) and larger than the upper limit of the range (above).

These graphics were designed according to [28]. Through them it is possible to verify that for the different *μ*_*t*_ scenarios, the analyzed intervals are approximately symmetrical around the true value of *β*_2_. We further note that for *μ*_*t*_ ∈ (0.02, 0.32), the intervals were better balanced when compared to the scenarios where *μ*_*t*_ ∈ (0.19, 0.86) and *μ*_*t*_ ∈ (0.78, 0.98). Overall, the bootstrap_*t*_ confidence interval stands out as better balanced. Fig 2 show that only the asymptotic confidence interval is approximately symmetric around the true value’s *γ*_2_.

The bootstrap_*t*_ confidence interval is slightly asymmetric around *γ*_2_. Regarding the bootstrap percentile confidence interval it exhibits very strong asymmetry, especially for the nominal 99% level. We also observe that the asymptotic confidence interval exhibits strong unbalancing, as the rates (% Right) are markedly higher than the observed rates (% Left). However, the bootstrap_*t*_ and percentile confidence intervals are approximately balanced for all nominal levels and scenarios. Therefore, based on the results presented, we suggest using the bootstrap_*t*_ confidence interval which showed better coverage and balance performances.

## 7 Application: Fluid Catalytic Cracking Data (FCC)

In this application the data are from the Chemistry Department of the National University of Colombia [29] and concerns a process regarding the volume and quality of gasoline produced in a refinery. The fluid catalytic cracking process known as Fluid Catalytic Cracking (FCC) is used to convert high molecular weight hydrocarbons into small molecules of higher commercial value by contacting them with a catalyst. This process is often described as the heart of the refinery, as it allows production to be tailored for a higher demand and especially high profit products [29]. The process catalyst consists of fine particles of 10 to 150 microns, easily fluidizable having the zeolite *Y* [29] as the main component. Another important substance that participates in the catalysis process is the vanadium. This chemical component is known to participate in catalyst destruction, reducing the active surface, selectivity and crystallinity of the zeolite *Y* especially in the presence of steam. Every 1000 ppm of vanadium in the catalyst is known to reduce gasoline yield by about 2.3%. The process also depends on the temperature, which must be close to 720° *C* [29]. The data set consists of 28 observations.

Aiming to fit a model to these data [13] chose the following candidate to covariates: steam (*x*_2_), temperature (*x*_3_) and vanadium concentration (*x*_4_). Moreover, the authors defined a linear predictor relating these covariates to unknown parameters. However, the residual analysis highlighted the possibility that the predictor is non-linear in some of the parameters. To build the nonlinear model, the authors follow several steps that are carefully detailed in their article. The model chosen uses probit and logarithmic link functions for the mean and dispersion submodels, respectively, and was defined as follows: $\Phi^{-1}\left(\mu_{t}\right)=\beta_{1}+\beta_{2}\frac{x_{t2}}{x_{t2}+\beta_{3}}+\beta_{4}x_{t3}+\beta_{5}\sqrt{x_{t4}}\;\text{and}\;\text{log}\left(\sigma_{t}^{2}\right)=\gamma_{1}+\gamma_{2}x_{t4}^{2},$, with *t* = 1, …, 28. Hereafter we shall so-called this model as ‘Model-I’. We emphasize that this simplex model outperformed a competing beta model [13]. Table 10 displays the maximum likelihood estimates, the bootstrap bias-corrected estimates, their respective standard errors (SE), and the *p*-values associated with the *Z*-tests for the significance of the model parameters. The ML estimates and their bootstrap corrected versions are quite similar with regards to the parameters of the mean submodel. Whereas the corrected estimates have lower standard errors than the ML estimates, with the exception only for the parameter *β*_1_. Concerning the parameters of the dispersion submodel, we notice that the maximum likelihood estimates and their corrected version present slightly different values. Additionally, their respective standard errors are quite similar.

**Table 10:** Maximum likelihood estimates $\left(\hat{\theta }\right)$, bootstrap bias-corrected estimates ($\bar{\theta }$), standard errors (SE) and *p*-values associated with *Z*-tests for the parameters of the [13]. Fluid Catalytic Cracking Data (FCC).

Parameters	*β*_1_	*β*_2_	*β*_3_	*β*_4_	*β*_5_	*γ*_1_	*γ*_2_
$\hat{\theta }$	1.399	−0.061	−27.843	−0.182	−0.420	0.756	−1.150
$\bar{\theta }$	1.411	−0.069	−27.782	−0.180	−0.428	0.898	−0.944
$EP(\hat{\theta })$	0.077	0.023	3.879	0.063	0.075	0.370	0.314
$EP(\bar{\theta })$	0.082	0.018	3.185	0.044	0.066	0.370	0.314
*P*-valor	<0.0001	0.007	<0.0001	0.004	<0.0001	0.041	0.0003

In Table 11 are reported the interval estimates of the Model-I parameters assuming nominal levels equal to 90%, 95% and 99%. Mindful the three estimation methods, ML-I_*a*_, Boot_*t*_ and Boot*p* it is notice that the interval estimates for *β*_1_, *β*_2_, *β*_4_ and *β*_5_ are quite similar. Whereas for the *β*_3_ parameter the bootstrap_*t*_ scheme estimates display lengths substantially longer than that of its competitors, for all nominal levels. Exemplify, for 1 − *α* = 0.95, the ML-I_*a*_, Boot_*t*_ and Boot*p* interval estimates are, respectively, (−35.445; −20.240), (−40.929; −9.544) and (−34.745; −20.474). Another feature concerning the bootstrap_*t*_ interval estimator is that some of its estimates include the value zero for the parameters. This fact occurs for the *β*_2_, (99%), (−0.466;0.142) and for the *β*_4_, (95%) and (99%). Nevertheless, *β*_2_ = 0 implies both in the exclusion of steam, which is a covariate recognized as important to the process and in the assumption of a linear predictor for the mean submodel. The most important information that the figures in Table 11 reveal, though, is that only the bootstrap_*t*_ interval considers the possibility that both *γ*_1_ and *γ*_2_ are simultaneously at equal to zero, both to 95% and 99%. Boot*p* reaches this conclusion for the 99% level, whereas for ML-I*a* estimator it is only possible that *γ*_1_ = 0 and when 1 − *α* = 0.99.

**Table 11:** ML-I_*a*_, Boot_*t*_ and Boot*p* interval estimates for the parameters of the Model ‘Model-I’. Fluid catalytic cracking (FCC) data.

90%			
Parameters	ML-I_*a*_	Boot_*t*_	Boot*p*
*β*_1_	(1.273; 1.526)	(1.235; 1.547)	(1.250; 1.531)
*β*_2_	(−0.098; −0.023)	(−0.117; −0.020)	(−0.086; −0.011)
*β*_3_	(−34.223; −21.463)	(−38.522; −15.849)	(−34.253; −21.156)
*β*_4_	(−0.285; −0.079)	(−0.346; −0.003)	(−0.308; −0.069)
*β*_5_	(−0.543; −0.297)	(−0.554; −0.268)	(−0.544; −0.284)
*γ*_1_	(0.148; 1.364)	(0.111; 1.790)	(−0.278; 1.401)
*γ*_2_	(−1.667; −0.633)	(−1.756; −0.191)	(−2.110; −0.545)
95%			
Parameters	ML-I_*a*_	Boot_*t*_	Boot*p*
*β*_1_	(1.249; 1.550)	(1.187; 1.580)	(1.186; 1.567)
*β*_2_	(−0.105; −0.016)	(−0.146; −0.008)	(−0.094; −0.004)
*β*_3_	(−35.445; −20.240)	(−40.929; −9.544)	(−34.745; −20.474)
*β*_4_	(−0.305; −0.059)	(−0.382; 0.061)	(−0.331; −0.038)
*β*_5_	(−0.567; −0.274)	(−0.579; −0.218)	(−0.566; −0.261)
*γ*_1_	(0.031; 1.481)	(−0.042; 1.992)	(−0.480; 1.554)
*γ*_2_	(−1.766; −0.534)	(−1.986; 0.001)	(−2.301; −0.315)
99%			
Parameters	ML-I_*a*_	Boot_*t*_	Boot*p*
*β*_1_	(1.201; 1.597)	(1.093; 1.613)	(1.142; 1.607)
*β*_2_	(−0.119; −0.002)	(−0.226; 0.018)	(−0.104; 0.010)
*β*_3_	(−37.834; −17.852)	(−46.445; −4.613)	(−37.023; −18.382)
*β*_4_	(−0.344; −0.020)	(−0.466; 0.148)	(−0.390; −0.013)
*β*_5_	(−0.613; −0.227)	(−0.660; −0.173)	(−0.620; −0.219)
*γ*_1_	(−0.196; 1.709)	(−0.244; 2.213)	(−0.701; 1.756)
*γ*_2_	(−1.960; −0.341)	(−2.316; 0.338)	(−2.639; 0.015)

Therefore, we shall evaluate a nonlinear simplex model with constant dispersion. Among the competing models, the one that presented the best goodness-of-fit uses log-log complementary and logarithmic link functions for the mean and dispersion submodel, as we shall describe in the following: $\text{log}\left(-\text{log}\left(1.0-\mu_{t}\right)\right)=\beta_{1}+\beta_{2}\frac{x_{t2}}{x_{t2}+\beta_{3}}+\beta_{4}x_{t3}+\beta_{5}\sqrt{x_{t4}}$ and $\text{log}\left(\sigma_{t}^{2}\right)=\gamma_{1}$, with *t* = 1, …, 28.

In what follows we shall appoint this model as ‘Model-II’ and provide some quantities about its parameters, namely: the maximum likelihood estimates, their bootstrap corrected versions: ‘(⋅)’ and the standard errors of the estimates ML: ‘[⋅]’. Thus, *β*_1_: 0.9112 (0.9051) [0.1081], *β*_2_: −1.4166 (−1.5062) [0.0016], *β*_3_: −26.1208 (−25.7744) [3.6902], *β*_4_: −0.2615 (−0.2571) [0.0635], *β*_5_: −0.3366 (−0.3431) [0.0607] and *γ*_1_: 0.0455 (0.2782) [0.2673]. Concerning Model-II it is possible to notice differences between the ML estimates and their bootstrap corrected versions. For example, ${\hat{\beta }}_{2}$ is equal to −1.42 while its corrected version becomes −1.51. A further important issue regarding Model-II is that the correct modeling of the dispersion, considerably reduced the standard errors of the estimates. The *SE* of ${\hat{\beta }}_{2}$ was 0.023 for Model-I whereas becomes 0.0016 for Model-II. Table 12 report the interval estimates of the ‘Model-II’ model parameters assuming nominal levels equal to 90%, 95% and 99%. It is noteworthy that the correct dispersion modeling improves the interval estimators performances. The accuracy of the boot_*t*_ and ML-I*a* interval estimators regarding to the *β*_2_ parameter is especially noteworthy. Let interval estimators be ML-I*a*, Boot_*t*_ and Boot*p*, respectively, the interval estimates are (−1.419; −1.413), (−1.419; −1.414) and (−1.904; −0.757), for 99%. For 95%, (−1.419; −1.413), (−1.419; −1.413) and (−2.032; −0.631). Finally, (−1.420; −1.412), (−1.4212; −1.4116) and (−2.3049; −0.3229), for 90% Here, we note that the Boot*p* displays a poor performance. It should be reminded that *β*_2_ is the parameter associated with the nonlinearity of the model, as well as *β*_3_. In fact, after dispersion was assumed constant the Boot_*t*_ scheme provided intervals for *β*_3_ with considerably shorter lengths compared to those of the ‘Model-I’ model.

**Table 12:** ML-I_*a*_, Boot_*t*_ and Boot*p* interval estimates for the parameters of Model-II. Fluid catalytic cracking (FCC) data.

90%			
Parameters	ML-I_*a*_	Boot_*t*_	Boot*p*
*β*_1_	(0.733;1.089)	(0.678;1.067)	(0.836;1.001)
*β*_2_	(−1.419; −1.413)	(−1.419; −1.414)	(−1.904; −0.757)
*β*_3_	(−32.190; −20.050)	(31.595; −18.054)	(−31.100; −22.146)
*β*_4_	(−0.365; −0.157)	(−0.383; −0.136)	(−0.376; −0.160)
*β*_5_	(−0.436; −0.236)	(−0.473; −0.221)	(−0.440; −0.216)
95%			
Parameters	ML-I_*a*_	Boot_*t*_	Boot*p*
*β*_1_	(0.699;1.123)	(0.641;1.113)	(0.817;1.017)
*β*_2_	(−1.419; −1.413)	(−1.419; −1.413)	(−2.032; −0.631)
*β*_3_	(−33.353; −18.888)	(−33.045; −15.755)	(−32.268; −21.152)
*β*_4_	(−0.385; −0.137)	(−0.410; −0.103)	(−0.410; −0.140)
*β*_5_	(−0.455; −0.217)	(−0.495; −0.197)	(−0.462; −0.200)
99%			
Parameters	ML-I_*a*_	Boot_*t*_	Boot*p*
*β*_1_	(0.632;1.189)	(0.4698;1.1882)	(0.7729;1.0600)
*β*_2_	(−1.420; −1.412)	(−1.4212; −1.4116)	(−2.3049; −0.3229)
*β*_3_	(−35.626; −16.615)	(−35.5726; −10.3756)	(−33.4200; −20.0357)
*β*_4_	(−0.425; −0.098)	(−0.4488; −0.0422)	(−0.4652; −0.1085)
*β*_5_	(−0.492; −0.180)	(−0.5163; −0.1802)	(−0.4823; −0.1633)

One area of research that we have been working on intensively regards model selection criteria for nonlinear models. The $R_{FC}^{2}$ criterion proposed by [7] for the beta regression model, defined as the square of the correlation between *g*(*y*) and ${\hat{\eta }}_{1}$ has proven quite effective in assessing the goodness-of-fit of models to data in the different applications we have performed on nonlinear models. The $R_{FC_{c}}^{2}$ corrected was proposed by [30] and is defined as $R_{{FC}_{c}}^{2}=1-\left(1-R_{FC}^{2}\right)\left(n-1\right)/\left(n-\left(k_{1}+q_{1}\right)\right)$. Models I and II display $\left\{R_{FC}^{2},R_{{FC}_{c}}^{2}\right\}$ measures equal to {0.6506, 0.5508} and {0.6818, 0.6095}, respectively. Thus, the choice of Model II is adequate and this model was inferred based on the bootstrap_*t*_ interval estimator.

## 8 Conclusion

In this paper we evaluate the point and interval estimation for the parameters indexing the nonlinear simplex regression model [13] in small samples. Additionally, we propose inferential improvements based on the bootstrap method.

Often MLEs can be biased when the sample size is small or even moderate. Thus, we consider comparing the point MLE performances of the model parameters and their corrected versions through a bootstrap scheme. The results of Monte Carlo simulations showed that, in general, the corrected estimators presented lower biases than the maximum likelihood estimators, evidencing the efficacy of the bootstrap scheme in bias correction. The MLEs of the parameters of the dispersion submodel are strongly biased, and the bootstrap corrected estimator provides a substantial reduction of these bias. Thus reinforcing the importance of using the proposed scheme in the bias correction of estimators of the nonlinear simplex regression model.

Usually the asymptotic confidence intervals based on MLE’s require large samples in order the coverage rates to be close to the nominal ones. An alternative to constructing adequate confidence intervals on small samples is through the bootstrap method. Thus, we consider three competing interval estimators, namely: the MLE-asymptotic, percentile and bootstrap_*t*_ estimator intervals. Regarding coverage rate in every simulation’s scenarios the bootstrap_*t*_ confidence interval outperformed the two others competitors. Furthermore, in almost all experiments it was the best balanced interval.

As a penalty for providing this outperformance, the bootstrap_*t*_ is typically larger than that of its competitors. Overall, however, the bootstap*t* interval proved to be the most appropriate estimator interval for nonlinear simplex regression. Not only from the simulation results, but it was also decisive in the application. In a scenario with only *n* = 28 observations, it was able to point the misspecification of the dispersion model which yield to a new and best fitted model.

## Supporting information

S1 File (OX)

S1 Text (TXT)
